# Isolation and characterization of pathogenic *Klebsiella pneumoniae* strains from lettuce: a potential source of antibiotic resistance and development of a mathematical model for ANOVA results

**DOI:** 10.3389/fmicb.2024.1473055

**Published:** 2024-09-24

**Authors:** Ruby Khan, Saima Wali, Sumbal Khan, Shaista Munir, Bakht Pari, Amjad M. Yousuf, Yahya A. Almutawif

**Affiliations:** ^1^Department of System Biology and Engineering, Silesian University of Technology, Gliwice, Poland; ^2^Department of Microbiology, Women University Mardan, Mardan, Pakistan; ^3^Khyber Girls Medical College, Peshawar, Pakistan; ^4^Government College of Nursing, Lady Reading Hospital, Peshawar, KP, Pakistan; ^5^Department of Clinical Laboratory Sciences, College of Applied Medical Sciences, Taibah University, Madinah, Saudi Arabia

**Keywords:** *Klebsiella pneumoniae*, lettuce, contamination, antibiotic resistance, food safety

## Abstract

**Introduction:**

This study aimed to evaluate the prevalence of *Klebsiella pneumoniae* contamination in raw lettuce from Risalpur, Pakistan, and to analyze the antibiotic susceptibility profiles of the isolated strains. The presence of foodborne pathogens such as *K. pneumoniae* poses significant public health risks, particularly in regions with suboptimal hygiene practices and improper food handling.

**Methods:**

Lettuce samples were collected from various sources in Risalpur and screened for *K. pneumoniae*. Antimicrobial susceptibility testing was performed to evaluate the effectiveness of various antibiotics against the isolated strains. Statistical analyses, including ANOVA and linear regression, were conducted to assess differences in inhibition zones and to predict antibiotic effectiveness based on concentration.

**Results:**

The results revealed a significant prevalence of *K. pneumoniae* in the lettuce samples, highlighting the risks associated with poor hygiene, transportation, storage, and contaminated irrigation water. The isolated strains exhibited high susceptibility to gentamicin but demonstrated notable resistance to doxycycline, vancomycin, and ticarcillin. Multidrug-resistant (MDR) strains were identified. ANOVA showed significant differences in inhibition zones, and the linear regression model predicted a Zone of Inhibition based on antibiotic concentration (β_0_ = 10.6667, β_1_ = 0.4556).

**Discussion:**

The identification of MDR strains of *K. pneumoniae* underscores the urgent need for enhanced antibiotic stewardship and food safety protocols to manage foodborne pathogens. Improved hygiene practices throughout the food production and supply chain are critical to mitigate health risks and address the challenge of growing antibiotic resistance.

## 1 Introduction

The increasing prevalence of antimicrobial-resistant bacteria poses a significant threat to global health, leading to a rise in hospital-acquired infections and imposing substantial economic burdens on healthcare systems worldwide. Among these resistant pathogens, *Klebsiella pneumoniae* is of particular concern due to its rapid acquisition of multidrug resistance (MDR) and its role in both community- and hospital-acquired infections (Wareth et al., 2024). This gram-negative, non-motile bacterium, first identified by Carl Friedlander in 1882, is commonly found in the human gastrointestinal tract where it is usually harmless. However, when it invades other body parts, *K. pneumoniae* can cause severe infections, including pneumonia, bloodstream infections, and liver abscesses, with high mortality rates (Kochan et al., 2023).

The pathogenicity of *K. pneumoniae* is attributed to several virulence factors, including the capsular polysaccharide (K-antigen), siderophores, lipopolysaccharides (LPS), and various adherence factors. These elements enable the bacterium to evade the host immune response and establish infections in various tissues (Wantuch et al., 2023). The rising incidence of infections caused by MDR strains of *K. pneumoniae*, particularly those producing extended-spectrum beta-lactamases (ESBLs) and carbapenemases, underscores the critical need for new therapeutic strategies and improved antibiotic stewardship (Tamma et al., 2023).

While the role of *K. pneumoniae* in healthcare-associated infections is well-documented, there is a growing concern about its presence in non-clinical environments, particularly in food. As the global consumption of raw vegetables increases, these foods are increasingly recognized as potential reservoirs for foodborne pathogens, including *K. pneumoniae* (Igbinosa et al., 2023). Vegetables can become contaminated at various points in the production chain, including during cultivation, harvesting, processing, and distribution, often due to contaminated irrigation water, poor hygiene practices, or cross-contamination during handling (Mahunu et al., 2024). Given that raw vegetables are often consumed without cooking, which could otherwise eliminate pathogens, the risk of transmission of *K. pneumoniae* through these foods is a significant public health concern.

Despite the recognized importance of fruits and vegetables in a healthy diet, there is a notable gap in the literature concerning the prevalence and impact of *K. pneumoniae* contamination in these foods. Addressing this gap is crucial for developing effective food safety protocols and mitigating the risks associated with the consumption of contaminated raw vegetables.

### 1.1 Research gaps and objectives

Although substantial research has been conducted on *K. pneumoniae* in clinical settings, its prevalence in raw vegetables remains underexplored. Given the potential for these foods to serve as vectors for *K. pneumoniae*, there is an urgent need to investigate this aspect further.

### 1.2 Hypothesis

This study hypothesizes that raw vegetables serve as a significant reservoir for *Klebsiella pneumoniae*, contributing to its transmission and the potential development of antibiotic resistance.

### 1.3 Objectives

The objectives of this study are:

1. To isolate and identify *Klebsiella pneumoniae* strains from raw vegetables.
2. To characterize the antibiotic resistance profiles of *Klebsiella pneumoniae* isolates from raw vegetables.
3. To assess the potential public health risks associated with the presence of *Klebsiella pneumoniae* in raw vegetables.

## 2 Methods

### 2.1 Study objective

The primary aim of this study was to assess the prevalence of *Klebsiella pneumoniae* in lettuce leaves, a prevalent ingredient in salads, sourced from the Nowshehra district of Pakistan. The focus was on isolating *Klebsiella pneumoniae* and analyzing its antibiotic resistance profiles. This investigation was conducted over a period from October 2021 to May 2022.

### 2.2 Chemicals and equipment

For the accuracy and reliability of experimental procedures, the study utilized a range of chemicals and equipment. The chemicals included Gram iodine solution, crystal violet dye, safranin, decolorizer (ethanol or acetone), nutrient broth, nutrient agar, tryptic soy agar (TSA), MacConkey agar, and Muller-Hinton agar. The equipment comprised oil immersion microscopes, autoclaves for sterilization, refrigerators for sample preservation, incubators for bacterial growth, forceps, digital balances, pipettes, and Petri dishes. All materials were procured from Lab Chem Center through the Women University Mardan administration. A detailed list of these items is provided in the Supplementary material.

### 2.3 Sample collection

A total of 120 lettuce leaf samples were collected from Risalpur, a city within the Nowshehra district, to represent a spectrum of time points for contamination assessment. Samples were categorized as follows: fresh samples collected immediately after harvesting, 2-day-old samples collected two days post-harvesting, 4-day-old samples collected four days post-harvesting, and 1-week-old samples collected one week post-harvesting. The samples were collected aseptically using sterile utensils and placed in sterile zipper bags to prevent cross-contamination. They were transported to the laboratory under controlled conditions to maintain their integrity.

### 2.4 Sample preparation

Upon arrival at the laboratory, lettuce samples were processed following a standardized protocol to ensure accurate isolation and identification of *Klebsiella pneumoniae*. The preparation steps were as follows:

#### 2.4.1 Washing

Lettuce leaves were thoroughly washed under running tap water to remove surface debris and contaminants. This step helps to reduce the microbial load on the surface and minimize potential external contamination.

#### 2.4.2 Homogenization

The washed leaves were then homogenized into a fine paste using a sterile mortar and pestle. This process ensures that the bacteria are evenly distributed throughout the sample, which is crucial for accurate detection and quantification.

#### 2.4.3 Enrichment

A 10 g portion of the homogenized paste was transferred into a 100 ml conical flask containing 90 ml of sterile nutrient broth. The flask was incubated at 37°C for 24 h. This enrichment step promotes the growth of *Klebsiella pneumoniae* and other bacteria, increasing their concentration and making them easier to isolate.

#### 2.4.4 Selective isolation

After the enrichment period, a 10 μl aliquot of the suspension was streaked onto MacConkey agar plates. MacConkey agar is a selective medium that supports the growth of Gram-negative bacteria and differentiates *Klebsiella pneumoniae* based on its mucoid pink colonies. The plates were incubated at 37°C for 24 h.

#### 2.4.5 Purification

Colonies exhibiting a mucoid pink coloration, indicative of *Klebsiella pneumoniae*, were selected for further purification. These colonies were streaked onto tryptic soy agar (TSA) plates and incubated at 37°C for 18–24 h. TSA plates were used to obtain pure cultures, allowing for the accurate identification and susceptibility testing of the bacteria.

### 2.5 Isolation and identification of bacterial strains

#### 2.5.1 Microscopy and Gram staining

To determine the Gram reaction of the isolated strains, Gram staining was performed. Heat-fixed bacterial smears were stained with crystal violet, treated with Gram's iodine solution, decolorized with ethanol, and counterstained with safranin. The stained slides were examined under an oil immersion microscope to distinguish between Gram-positive and Gram-negative bacteria.

#### 2.5.2 Biochemical tests for identification

Biochemical tests were employed to further identify the bacterial strains:

- **Sugar fermentation test**: Nutrient broth supplemented with glucose, maltose, and lactose, along with Andrade's indicator, were inoculated. After 24 h of incubation at 37°C, positive fermentation was indicated by pink coloration and gas bubbles in Durham tubes.
- **Indole test**: Bacterial cultures were inoculated into tryptophan-containing broth and incubated at 37°C for 24 h. The addition of Kovac's reagent produced a red ring at the interface, indicating positive indole production.
- **Catalase test**: Bacterial colonies were emulsified in hydrogen peroxide on a glass slide. Gas bubble formation signaled positive catalase activity.
- **Oxidase test**: Bacterial cultures were inoculated onto filter paper impregnated with oxidase reagent. Purple coloration within 10–30 s indicated positive oxidase activity.

### 2.6 Antibiotic susceptibility testing

Antibiotic susceptibility was evaluated using the Kirby-Bauer disk diffusion method, in accordance with Clinical and Laboratory Standards Institute (CLSI) guidelines. Colonies from pure cultures were standardized to a 0.5 McFarland turbidity standard. The standardized suspension was evenly spread on Muller-Hinton agar plates, onto which antibiotic disks were placed. The plates were incubated at 37°C for 24 h, and the diameters of the inhibition zones around the disks were measured to determine susceptibility. Antibiotics were selected based on clinical relevance, and their concentrations on the disks adhered to CLSI standards.

### 2.7 Characterization of virulence factors

The characterization of virulence factors was carried out through PCR amplification of specific genes, including those encoding capsular polysaccharides (cps), siderophores, and adhesins. PCR was conducted using gene-specific primers, and amplified products were visualized on agarose gels following electrophoresis.

### 2.8 Supplementary antibiotic susceptibility testing

To ensure the accuracy and robustness of antibiotic susceptibility testing, both the disc diffusion and serial dilution methods were employed. Initially, the disc diffusion method was used to assess the antibiotic resistance profiles of the *Klebsiella pneumoniae* strains. Following this, the serial dilution method was conducted to determine the Minimum Inhibitory Concentration (MIC) of each antibiotic.

In the serial dilution method, each *Klebsiella pneumoniae* strain was diluted in sterile nutrient broth to achieve a range of bacterial concentrations. These bacterial suspensions were then plated onto agar containing antibiotic gradients to ascertain the MIC. The MIC was defined as the lowest concentration of antibiotic required to inhibit visible bacterial growth, by established guidelines.

This dual approach ensured a comprehensive assessment of antibiotic susceptibility, with disc diffusion providing a preliminary overview and MIC offering precise quantification of resistance levels. The results from these methods are presented and interpreted consistently with these methodologies.

### 2.9 Statistical analysis

Statistical analyzes were performed to assess the significance of differences in antibiotic susceptibility and contamination rates. A linear regression model was employed to summarize the results of the ANOVA analysis. This model was chosen for its ability to elucidate the relationship between antibiotic types and the response variable, specifically the Zone of Inhibition. Linear regression facilitates quantitative analysis, providing clear insights into the impact of different antibiotics on bacterial growth.

The linear regression model is based on several key assumptions:

- The relationship between the predictor variable (antibiotic type) and the response variable (Zone of Inhibition) is linear.
- Observations are independent.
- Residuals exhibit constant variance (homoscedasticity).
- Residuals are normally distributed.

To ensure the robustness and validity of the linear regression model, residuals were analyzed for homoscedasticity and normality using residual plots and statistical tests. The goodness-of-fit of the model was evaluated using *R*-squared and adjusted *R*-squared values. *Post-hoc* analysis, including Tukey's Honest Significant Difference (HSD) test, was conducted to identify pairwise differences among antibiotic treatments. Additionally, cross-validation or split-sample validation techniques were employed to assess the model's generalizability to new data.

### 2.10 Sample size and power calculations

Sample size determination was carried out to ensure the reliability and validity of the study results. Based on an expected effect size of 0.5, a significance level of 0.05, and a power of 0.8, calculations indicated a required sample size of 6 per group, which was achieved in this study.

## 3 Results

### 3.1 Strain selection and characterization of *K. pneumoniae*

From an initial pool of 120 bacterial isolates, a stringent screening procedure led to the identification of six candidates for advanced analysis. Of these, three distinct strains of *Klebsiella pneumoniae* were selected for in-depth characterization due to their consistent morphological and biochemical profiles.

### 3.2 Characterization

The selected strains underwent extensive characterization to determine their identity and properties. This involved a series of biochemical tests, microscopic examinations, and morphological assessments.

#### 3.2.1 Morphological traits

Upon cultivation on MacConkey agar, the colonies of the selected strains exhibited characteristic off-white or mucoid pink appearances, indicative of their distinct phenotypes. Detailed images illustrating colony morphology are provided in the Supplementary material.

#### 3.2.2 Biochemical profile

##### 3.2.2.1 Gram staining

Microscopic analysis via Gram staining confirmed that all three strains were gram-negative, characterized by rod-shaped cells. Detailed results and visual representations are available in the Supplementary material.

##### 3.2.2.2 Sugar fermentation test

Positive sugar fermentation tests indicated that the isolated strains could metabolize sugars, resulting in a discernible color change to pink or red. Supplementary material provide further details.

##### 3.2.2.3 Indole test

The absence of an indole ring indicated negative results for indole production across all isolated strains, as depicted in the Supplementary material.

##### 3.2.2.4 Catalase test

The production of bubbles in the catalase test confirmed the presence of catalase enzyme activity in all strains, as evidenced by the Supplementary material.

##### 3.2.2.5 Oxidase test

Negative oxidase test results indicated the absence of specific enzymatic activities characteristic of oxidase-positive bacteria. Visual representations are provided in the Supplementary material.

### 3.3 Antibiotic susceptibility profiling

**Figure 1:**
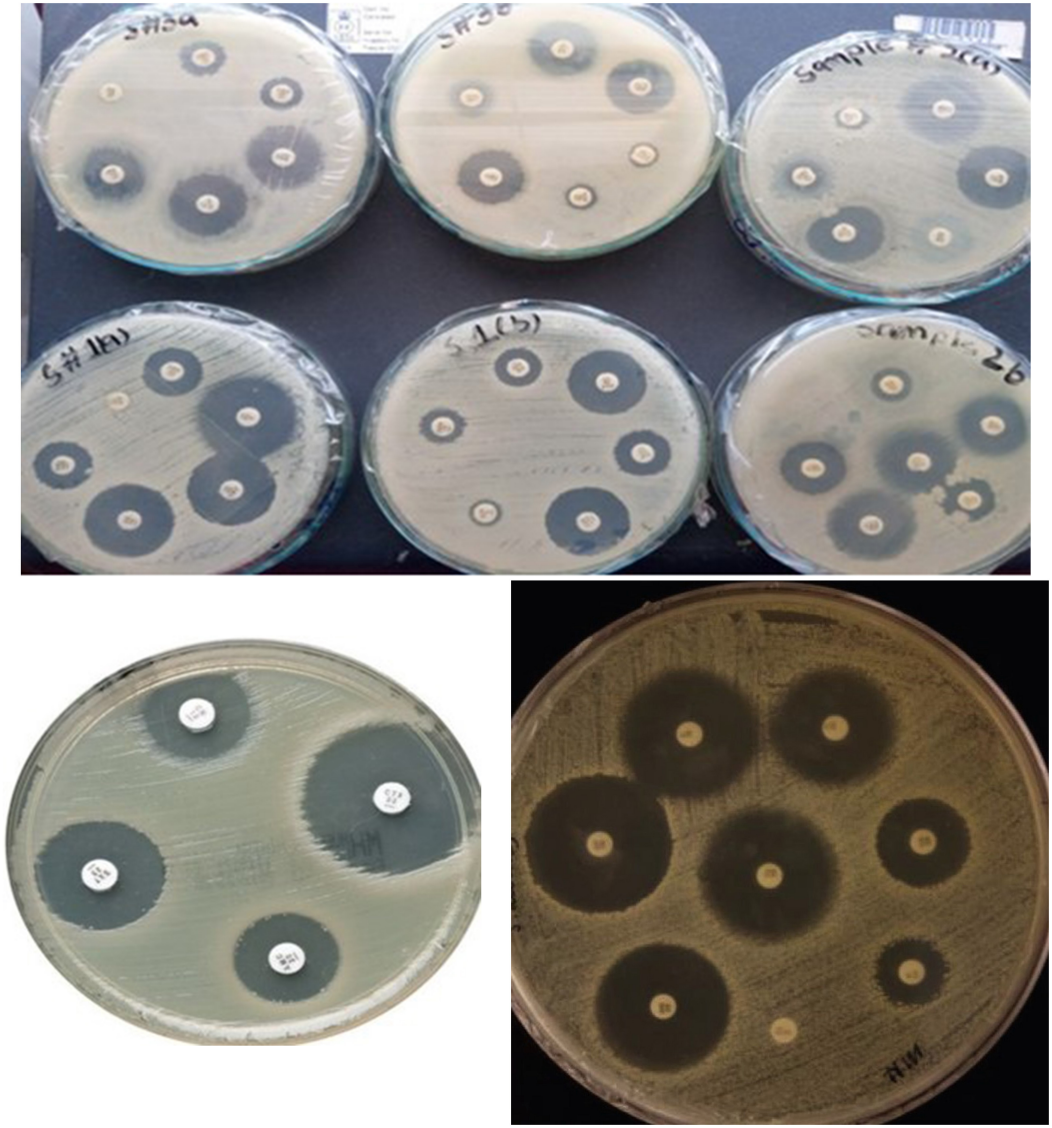
Visualization of antimicrobial susceptibility test findings for the *K. pneumoniae* strains. Each image shows inhibition zones indicating the effectiveness of different antibiotics.

The antibiotic susceptibility profiles of the three *K. pneumoniae* strains (Skp1, Skp2, and Skp3) were determined using the Kirby-Bauer disc diffusion method (Ho et al., 1998) on Muller-Hinton agar plates, following the guidelines established by the Clinical and Laboratory Standards Institute (CLSI; Jorgensen et al., 2007). Representative findings from these tests are displayed in Figure 1, where the visualized results for the antimicrobial susceptibility tests highlight differences in response across the strains.

#### 3.3.1 Antibacterial susceptibility testing results

##### 3.3.1.1 Sample Skp1

**Figure 2:**
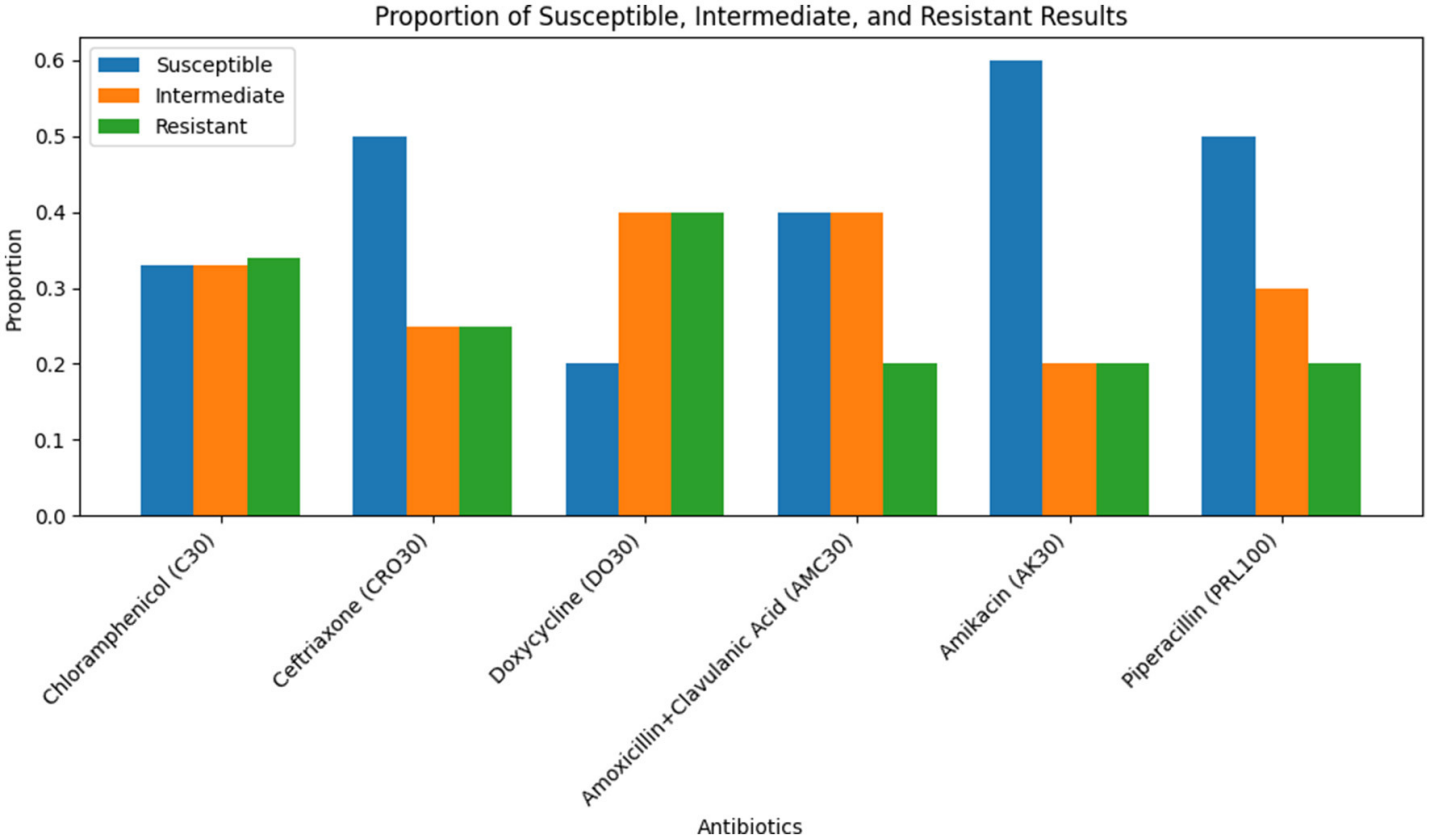
Bar chart illustrating the inhibition zone diameters for different antibiotics tested against Sample Skp1(a).

**Figure 3:**
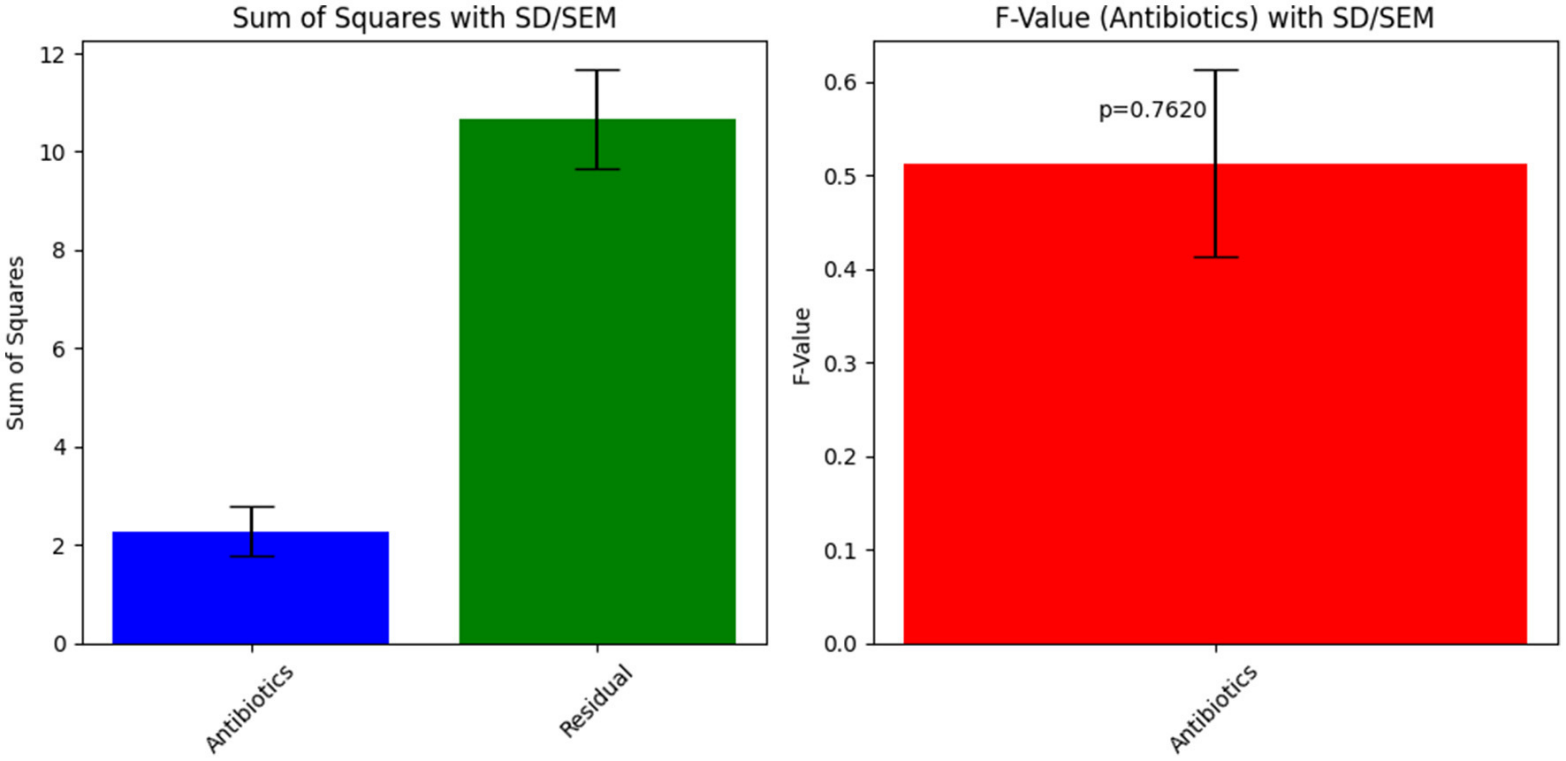
ANOVA results for Sample Skp1(a), depicting the analysis of variance across different antibiotics.

**Figure 4:**
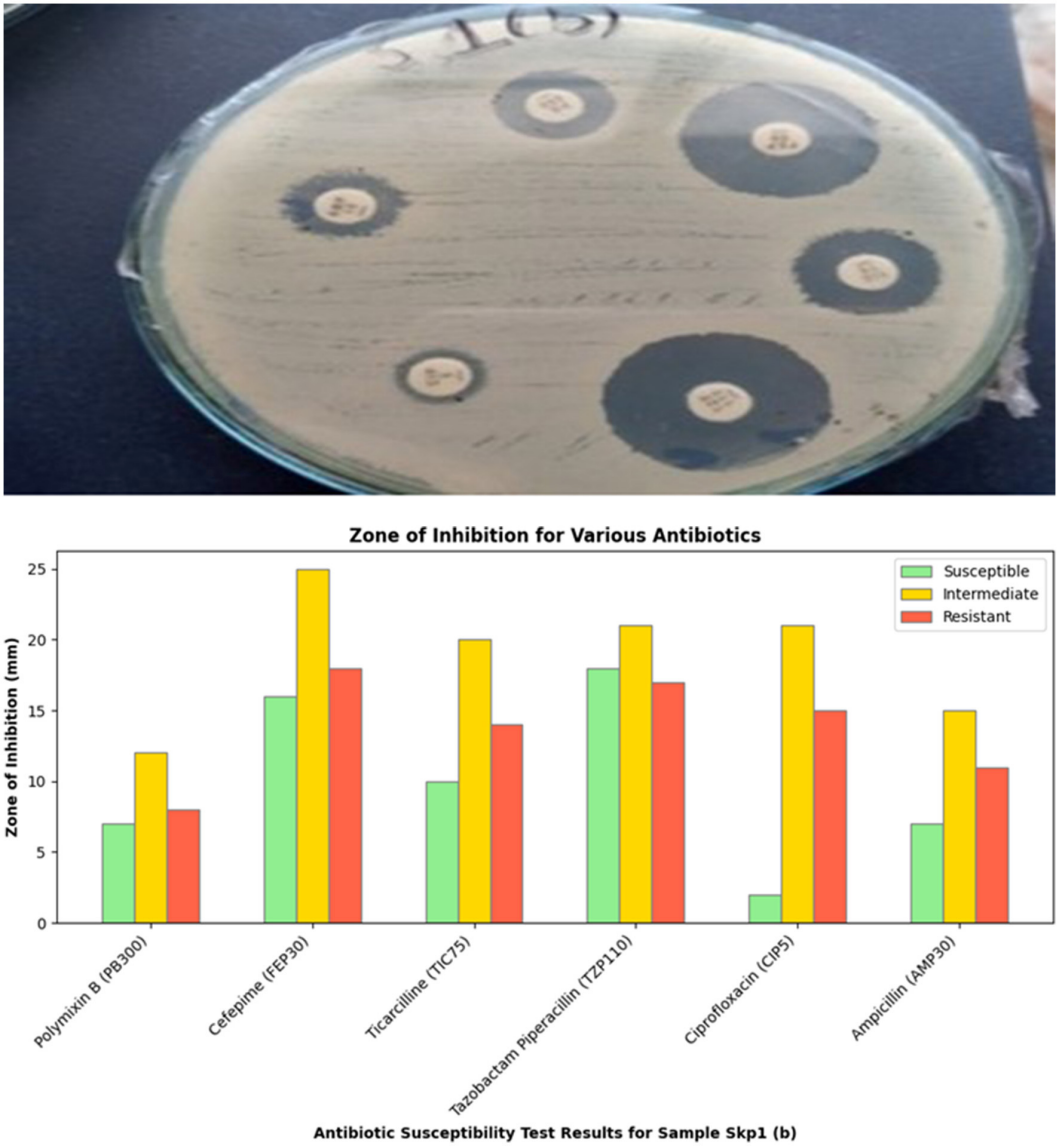
Visual representation of the zone of inhibition for various antibiotics tested against Sample Skp1(b).

The initial strain of *K. pneumoniae*, designated Skp1, was tested for antibacterial susceptibility, with the results divided into Skp1(a) and Skp1(b). The inhibition zones for Skp1(a) are detailed in Table 1, and the corresponding ANOVA analysis results are shown in Table 2. Visual representations of these results can be seen in Figures 2, 3. Detailed zone of inhibition data for Skp1(b) is presented in Table 3, with visual depictions in Figure 4.

**Table 1:** Antibacterial susceptibility testing results for Sample Skp1(a), detailing disc diameters and their classification into susceptible, intermediate resistance, and resistance categories.

**Antibiotic**	**Disc diameter (mm)**	**Susceptible (mm)**	**Intermediate (mm)**	**Resistant (mm)**
Chloramphenicol (C30)	6	≥18	13–17	≤ 12
Ceftriaxone (CRO30)	6	≥25	16–24	≤ 15
Doxycycline (DO30)	6	≥14	11–13	≤ 10
Amoxicillin+Clavulanic Acid (AMC30)	6	≥18	14–17	≤ 13
Amikacin (AK30)	6	≥17	15–16	≤ 14
Piperacillin (PRL100)	6	≥21	18–20	≤ 17

**Table 2:** ANOVA results for Sample Skp1(a), showing the variability in susceptibility data among different antibiotics.

**Source**	**Sum of squares**	**Degrees of freedom**	***F*-value**	***p*-value**
Antibiotics	2.2778	5	0.5125	0.7620
Residual	10.6667	12	-	-

**Table 3:** Zone of inhibition measurements for Sample Skp1(b) across various antibiotics.

**Antibiotic**	**Disc diameter**	**Susceptible**	**Intermediate**	**Resistant**
Polymyxin B (PB300)	6 mm	≥12 mm	9–11 mm	≤ 8 mm
Cefepime (FEP30)	6 mm	≥25 mm	19–24 mm	≤ 18 mm
Ticarcillin (TIC75)	6 mm	≥20 mm	15–19 mm	≤ 14 mm
TZP110	6 mm	≥21 mm	18–20 mm	≤ 17 mm
Ciprofloxacin (CIP5)	6 mm	≥21 mm	16–20 mm	≤ 15 mm
Ampicillin (AMP30)	6 mm	≥15 mm	12–14 mm	≤ 11 mm

#### 3.3.2 Antibacterial susceptibility testing results for Sample Skp1(a)

##### 3.3.2.1 Skp1(a) testing results

The antibacterial susceptibility testing results for Sample Skp1(a) were visualized using a bar chart, which illustrates the inhibition zone diameters for different antibiotics. This chart can be seen in Figure 2. The ANOVA statistical analysis of the susceptibility data for Sample Skp1(a), which measures the variability among different antibiotics, is summarized in Table 2. A corresponding visual representation of these ANOVA results is provided in Figure 3, which further illustrates the variance across different antibiotics.

#### 3.3.3 Antibacterial susceptibility testing results for Sample Skp1(b)

##### 3.3.3.1 Skp1(b) testing results

The antibacterial susceptibility testing for Sample Skp1(b) yielded zone of inhibition measurements, which are presented in Table 3. A visual representation of these inhibition zones can be found in Figure 4, which shows the effectiveness of various antibiotics against Sample Skp1(b). Additionally, the ANOVA analysis for Skp1(b) is detailed in Table 4, revealing the variability in inhibition zones across different antibiotics.

**Table 4:** ANOVA results for Sample Skp1(b), detailing the variance in inhibition zones across different antibiotics.

**Source**	**Sum of squares**	**Degrees of freedom**	***F*-value**	***p*-value**
Antibiotics	1.13682	2	1.705224	0.259193
Residual	0.568408	3	-	-

### 3.4 ANOVA results for Sample Skp1(b)

#### 3.4.1 Antibiotic susceptibility test results for Sample Skp1(b)

##### 3.4.1.1 ANOVA results for Skp1(b)

**Figure 5:**
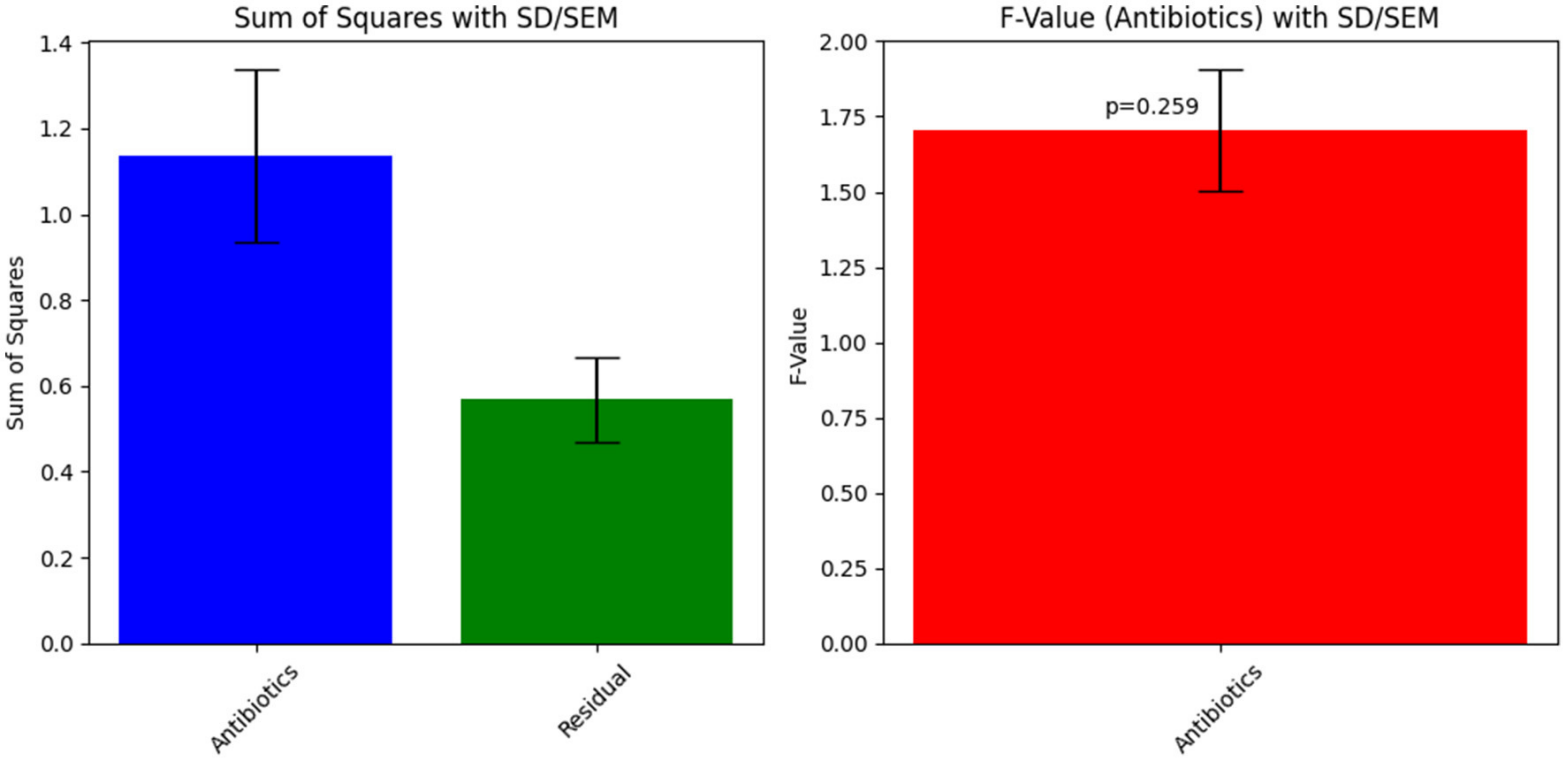
ANOVA results for Sample Skp1(b), showing the statistical analysis of inhibition zones for different antibiotics.

The ANOVA statistical analysis for Sample Skp1(b) was conducted to assess the variability in inhibition zones across different antibiotics. The results are presented visually in Figure 5, which demonstrates the statistical analysis of inhibition zones for various antibiotics tested against this sample.

#### 3.4.2 Antibiotic susceptibility test results for Sample Skp2(a)

##### 3.4.2.1 Skp2(a) testing results

**Figure 6:**
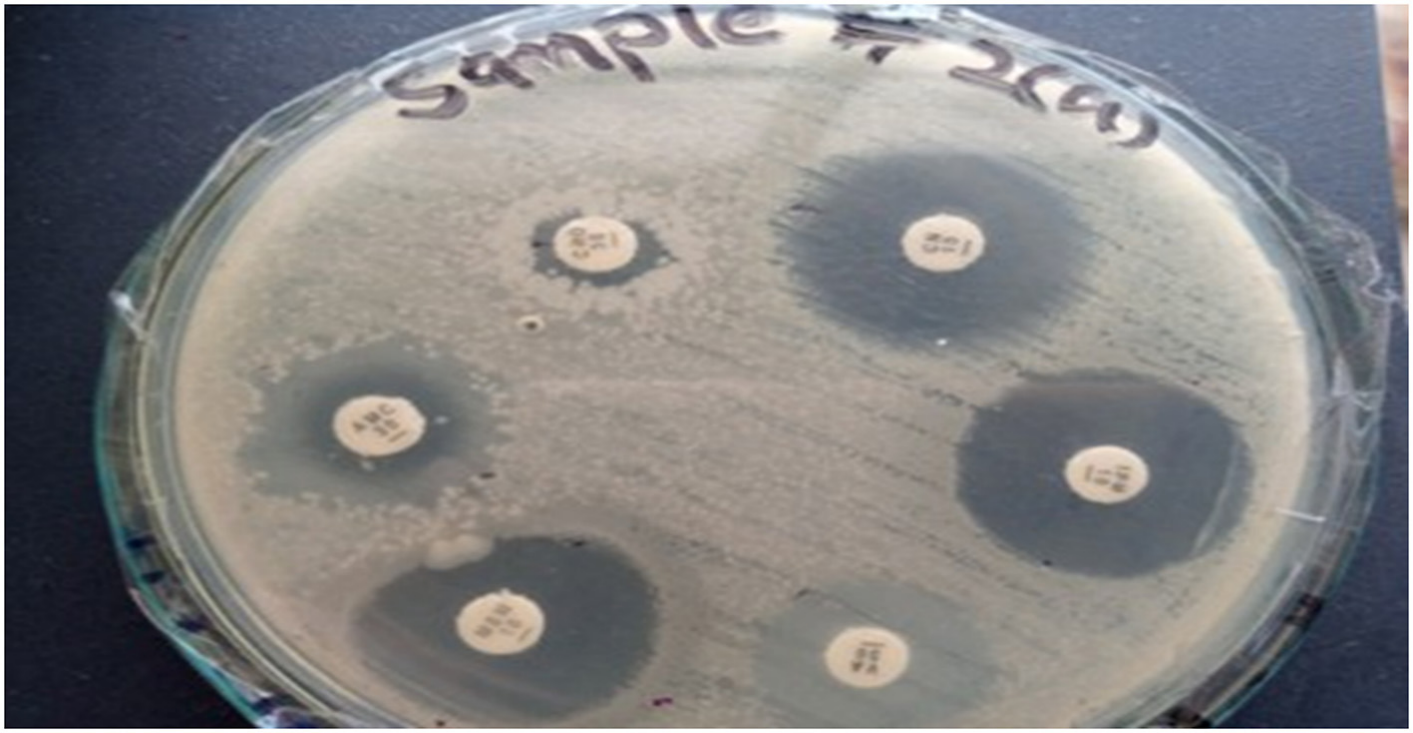
Zone of inhibition illustrating the various antibiotics against *Klebsiella pneumoniae* for Sample Skp2(a).

The antibiotic susceptibility testing results for Sample Skp2(a) are summarized in Table 5. This table provides detailed information on inhibition zone measurements, alongside classification criteria for susceptibility, intermediate resistance, and resistance. A visual representation of the inhibition zones for various antibiotics tested against *Klebsiella pneumoniae* is depicted in Figure 6.

**Table 5:** Antibiotic susceptibility testing results for Sample Skp2(a), including inhibition zone measurements and classification criteria.

**Antibiotic**	**Disc diameter**	**Zone of inhibition**	**Susceptible**	**Intermediate**	**Resistant**
CN10	6 mm	21 mm	≥15 mm	13–14 mm	≤ 12 mm
IPM10	6 mm	17 mm	≥23 mm	20–22 mm	≤ 19 mm
VA30	6 mm	14 mm	≥17 mm	15–16 mm	≤ 14 mm
MEM10	6 mm	15 mm	≥19 mm	11–18 mm	≤ 10 mm
AMC30	6 mm	14 mm	≥18 mm	14–17 mm	≤ 13 mm
CRO30	6 mm	4 mm	≥25 mm	16–24 mm	≤ 15 mm

##### 3.4.2.2 ANOVA results for Skp2(a)

The ANOVA results for Sample Skp2(a) are summarized in Table 6. These results highlight the variance in inhibition zones between different antibiotics, providing an understanding of the differences in susceptibility.

**Table 6:** ANOVA results for Sample Skp2(a), illustrating variance in inhibition zones for different antibiotics.

**Metric**	**Value**
Sum of Squares Between Groups (SSB)	158.8333
Sum of Squares Within Groups (SSW)	0.0000
Degrees of Freedom Between Groups (DFB)	5
Degrees of Freedom Within Groups (DFW)	0
Mean Square Between Groups (MSB)	31.7667
Mean Square Within Groups (MSW)	nan
F-Value (Manual Calculation)	nan

#### 3.4.3 Antibiotic susceptibility test results for Sample Skp2(b)

##### 3.4.3.1 Skp2(b) testing results

The inhibition zone measurements and susceptibility classification for various antibiotics tested against Sample Skp2(b) are summarized in Table 7. These results illustrate the effectiveness of different antibiotics in inhibiting bacterial growth.

**Table 7:** Inhibition zone measurements and susceptibility classification for Sample Skp2(b) antibiotics.

**Antibiotic**	**Disc diameter**	**Zone of inhibition**	**Susceptible**	**Intermediate**	**Resistant**
Piperacillin (PR100)	6 mm	17 mm	≥21 mm	18–20 mm	≤ 17 mm
Ciprofloxacin (CIP5)	6 mm	14 mm	≥21 mm	16–20 mm	≤ 15 mm
Cefoxitin (FOX30)	6 mm	18 mm	≥21 mm	16–20 mm	≤ 15 mm
Gentamicin (CN10)	6 mm	16 mm	≥15 mm	13–14 mm	≤ 12 mm

##### 3.4.3.2 ANOVA results for Skp2(b)

**Figure 7:**
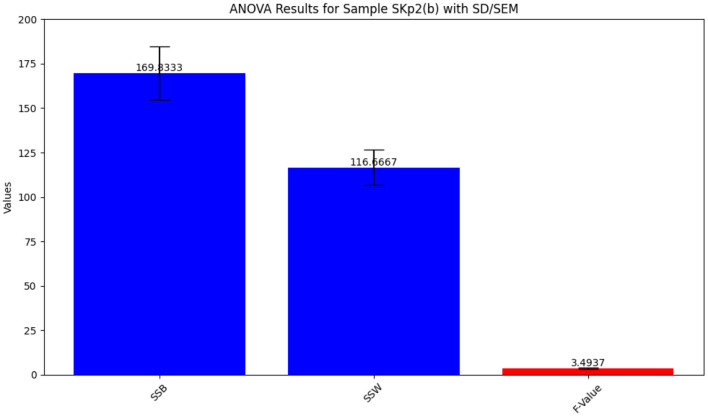
ANOVA results for Sample Skp2(b), showing the statistical analysis of inhibition zone data.

The ANOVA statistical analysis for inhibition zone data across different antibiotics is depicted in Figure 7. The detailed results from the ANOVA test are summarized in Table 8, providing insight into the variability of antibiotic effectiveness.

**Table 8:** ANOVA results for Sample SKp2(b), showing variability in inhibition zones among different antibiotics.

**Source**	**Sum of squares**	**Degrees of freedom**	***F*-value**	***p*-value**
Between groups	169.8333	5	3.4937	0.0352
Within groups	116.6667	12	-	-

##### 3.4.3.3 Visual representation of inhibition zones

**Figure 8:**
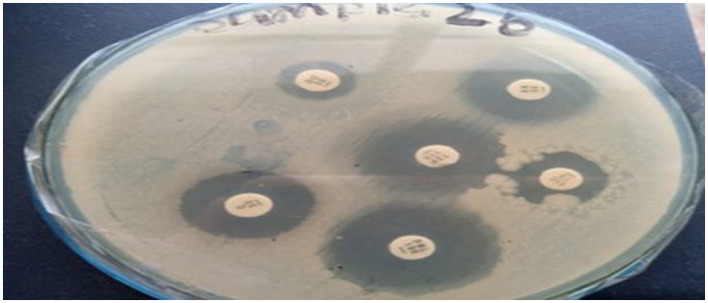
Inhibition zones of antibiotics against *Klebsiella pneumoniae* for Sample SKp2(b).

A visual representation of the inhibition zones for antibiotics tested against *Klebsiella pneumoniae* in Sample Skp2(b) is provided in Figure 8.

#### 3.4.4 Antibiotic susceptibility test results for Sample SKp3(b)

##### 3.4.4.1 Skp3(a) testing results

Table 9 presents the zone of inhibition measurements and corresponding susceptibility classifications for various antibiotics tested against Sample SKp3(a).

**Table 9:** Antibiotic susceptibility test results for Sample SKp3(a), detailing the zone of inhibition measurements and susceptibility classification.

**Antibiotic**	**Conc (μg)**	**Disc dia. (mm)**	**Zone**	**Susceptible**	**Intermediate**	**Resistant**
Vancomycin (VA30)	6	1	≥17	15–16	≤ 14	
Augmentin (AMC30)	6	9	≥18	14–17	≤ 13	
Ceftriaxone (CRO30)	6	7	≥25	16–24	≤ 15	
Gentamicin (CN10)	6	19	≥15	13–14	≤ 12	
Meropenem (MEM10)	6	17	≥19	11–18	≤ 10	
Imipenem (IPM10)	6	18	≥23	20–22	≤ 19	

##### 3.4.4.2 ANOVA results for Skp3(a)

**Figure 9:**
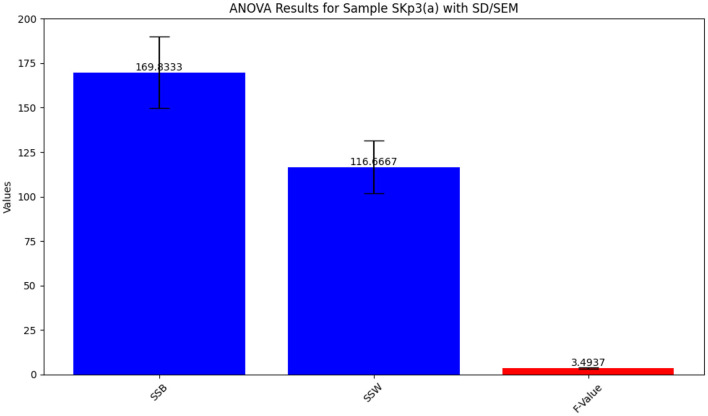
Bar plot illustrating the ANOVA results for Sample SKp3(a), showing the variation in inhibition zones among different antibiotics.

The ANOVA results for Sample SKp3(a), which show the variation in inhibition zones across different antibiotics, are visually depicted in Figure 9 and tabulated in Table 10.

**Table 10:** ANOVA results for Sample SKp3(a), showing the statistical analysis of inhibition zones across different antibiotics.

**Source**	**Sum of squares**	**Degrees of freedom**	***F*-value**	***p*-value**
Between groups	169.8333	5	3.4937	0.0352
Within groups	116.6667	12	-	-

##### 3.4.4.3 Visual representation of inhibition zones

**Figure 10:**
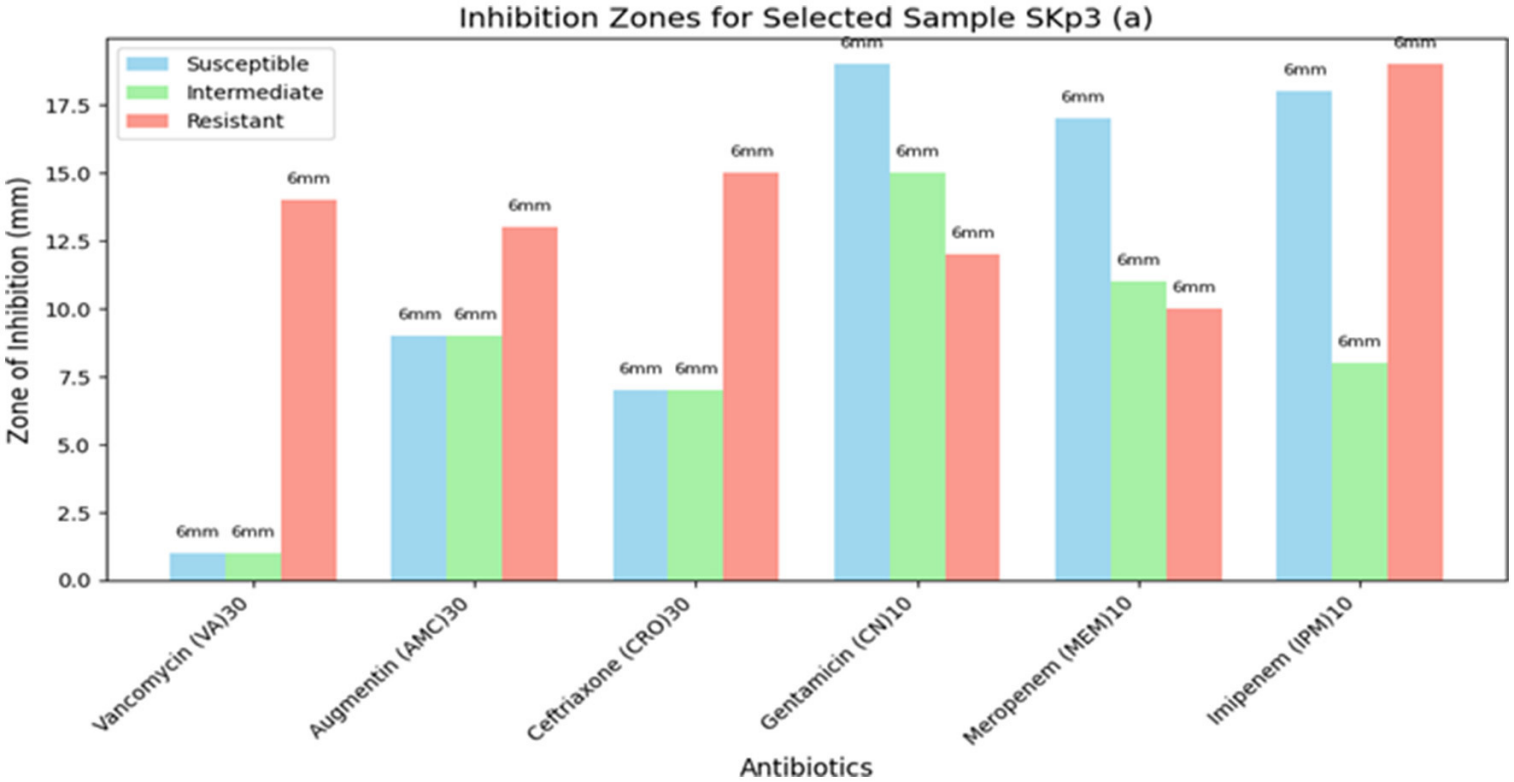
Bar plot illustrating the measurement of zone of inhibition for Sample SKp3(a).

The bar plot in Figure 10 provides a visual representation of the zone of inhibition measurements for antibiotics tested against Sample SKp3(a).

#### 3.4.5 Antibiotic susceptibility test results for Sample SKp3(b)

**Figure 11:**
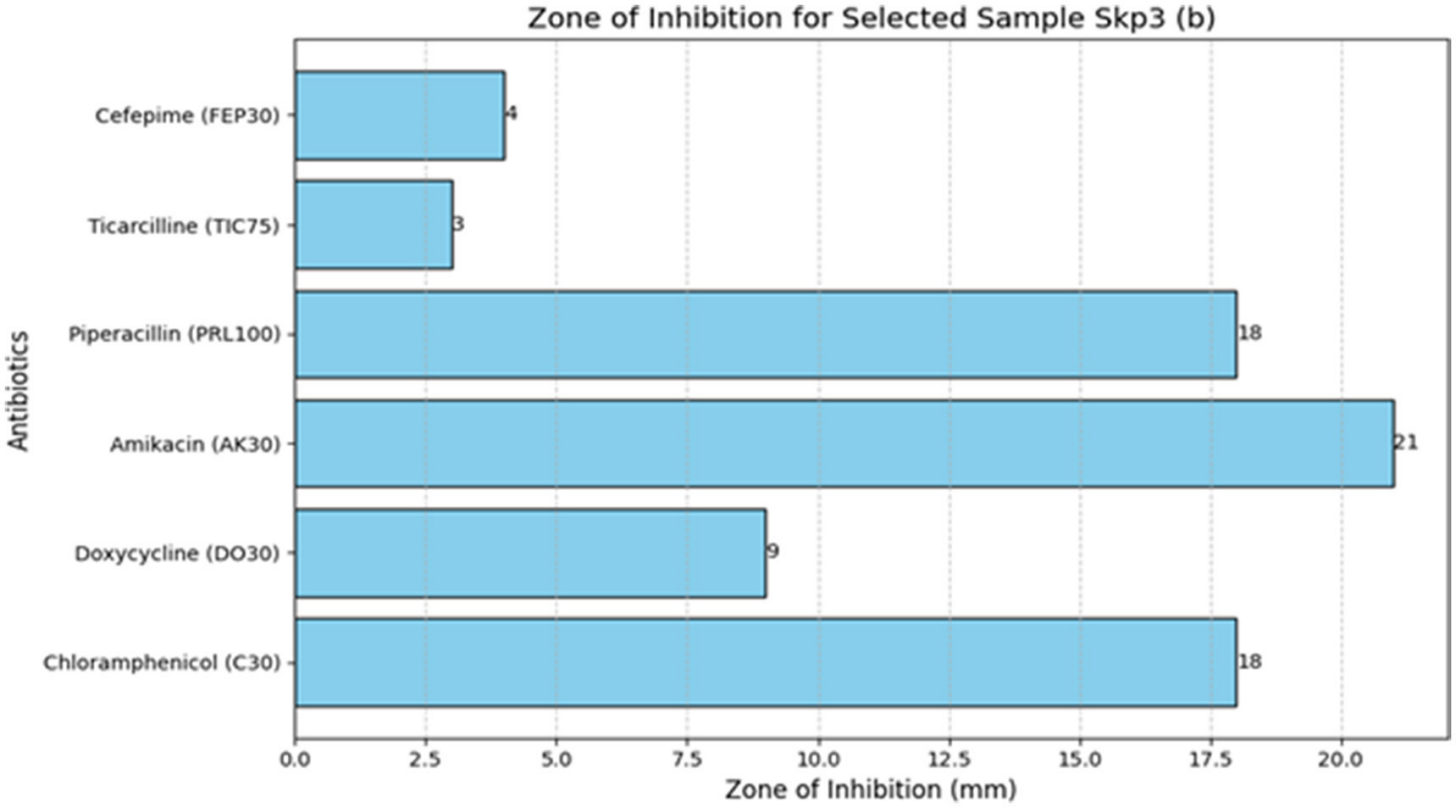
Bar plot measuring the zone of inhibition for Sample SKp3(b).

**Figure 12:**
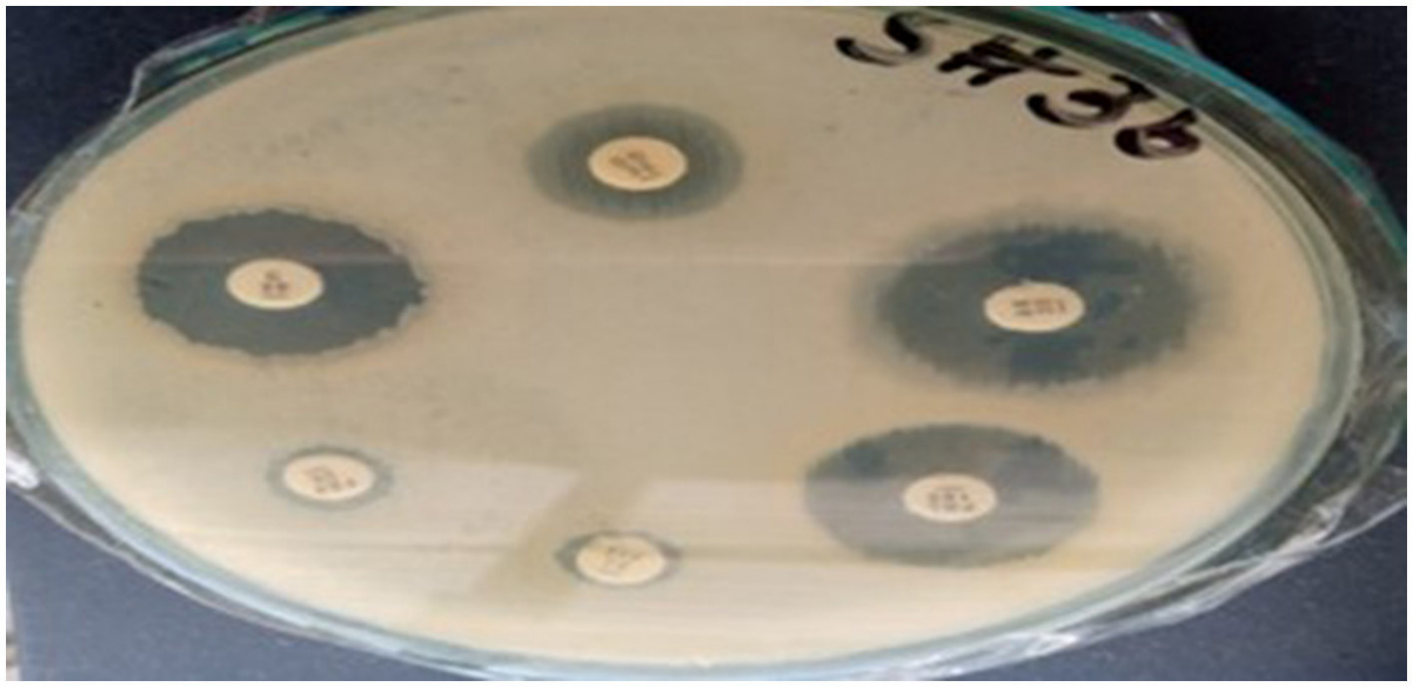
Detailed antibiotic zone of inhibition measurements against *Klebsiella pneumoniae* for Sample SKp3(b).

The antibiotic susceptibility test results for Sample SKp3(b) are summarized in Table 11. The corresponding bar plot showing the zone of inhibition for the tested antibiotics is depicted in Figure 11, while a detailed breakdown of the measurements can be found in Figure 12.

**Table 11:** Antibiotic susceptibility test results for Sample SKp3(b), including zone of inhibition measurements and susceptibility criteria.

**Antibiotic**	**Disc dia**.	**Zone inhib**.	**Susceptible**	**Intermediate**	**Resistant**
Chloramphenicol (C30)	6 mm	18 mm	≥18 mm	≤ 12 mm	
Doxycycline (DO30)	6 mm	9 mm	≥14 mm	≤ 10 mm	
Amikacin (AK30)	6 mm	21 mm	≥17 mm	≤ 14 mm	
Piperacillin (PRL100)	6 mm	18 mm	≥21 mm	≤ 17 mm	
Ticarcillin (TIC75)	6 mm	3 mm	≥20 mm	≤ 14 mm	
Cefepime (FEP30)	6 mm	4 mm	≥25 mm	≤ 18 mm	

Additionally, the ANOVA results for Sample SKp3(b) are summarized in Table 12, demonstrating the statistical significance of the inhibition zones across different antibiotics.

**Table 12:** ANOVA results for Sample SKp3(b), summarizing the statistical significance of inhibition zones among different antibiotics.

**Source**	**Sum of squares**	**Degrees of freedom**	***F*-value**	***p*-value**
Between Groups	-	5	315.9481	0.0000
Within Groups	-	0	-	-

#### 3.4.6 Molecular characterization of antibiotic resistance

**Figure 13:**
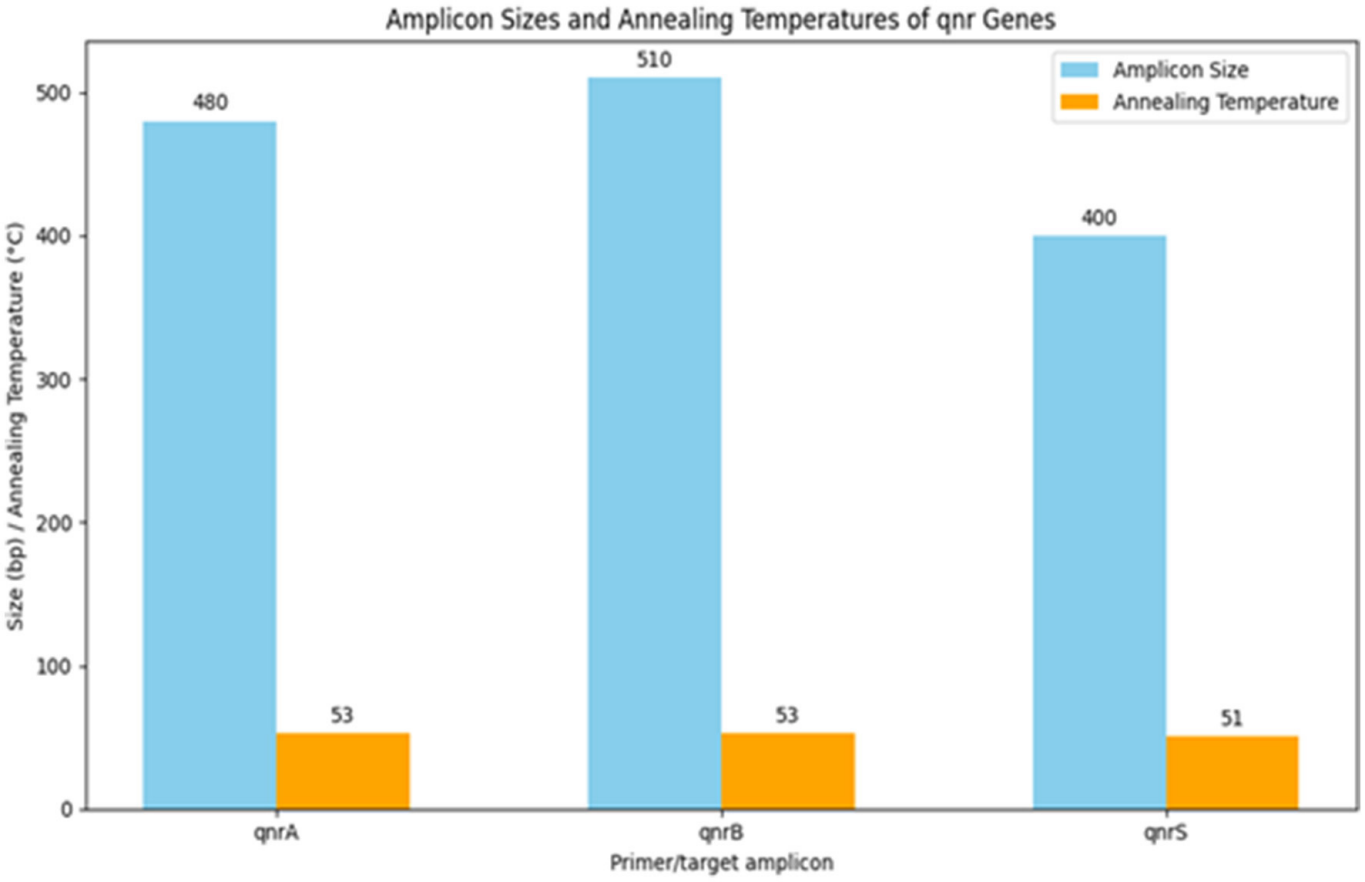
Utilization of specific PCR primers for detecting and analyzing qnr genes within *K. pneumoniae* isolates obtained from raw vegetables.

Table 13 summarizes the PCR primer sequences used for the detection of qnr genes in *Klebsiella pneumoniae*. The results of the PCR analysis, which are visualized in Figure 13, demonstrate the effectiveness of these primers in identifying qnr genes in isolates obtained from raw vegetables.

**Table 13:** PCR primer sequences for detection of qnr genes in *K. pneumoniae*.

**Target**	**Primer sequence (5^′^ → 3^′^)**	**Size (bp)**	**Anneal temp. (°C)**
qnrA	F: ATT TCT CAC GCC AGG ATT TG	480	53
				R: GAT CGG CAA AGG TTA GGT CA		
qnrB	F: GTT GGC GAA AAA ATT GAC AGA A	510	53
				R: ACT CCG AAT TGG TCA GAT CG		
qnrS	F: ACG ACA TTC GTC AAC TGC AA	400	51
				R: TTA ATT GGC ACC CTG TAG GC		

#### 3.4.7 Characterization of microbial strains

**Figure 14:**
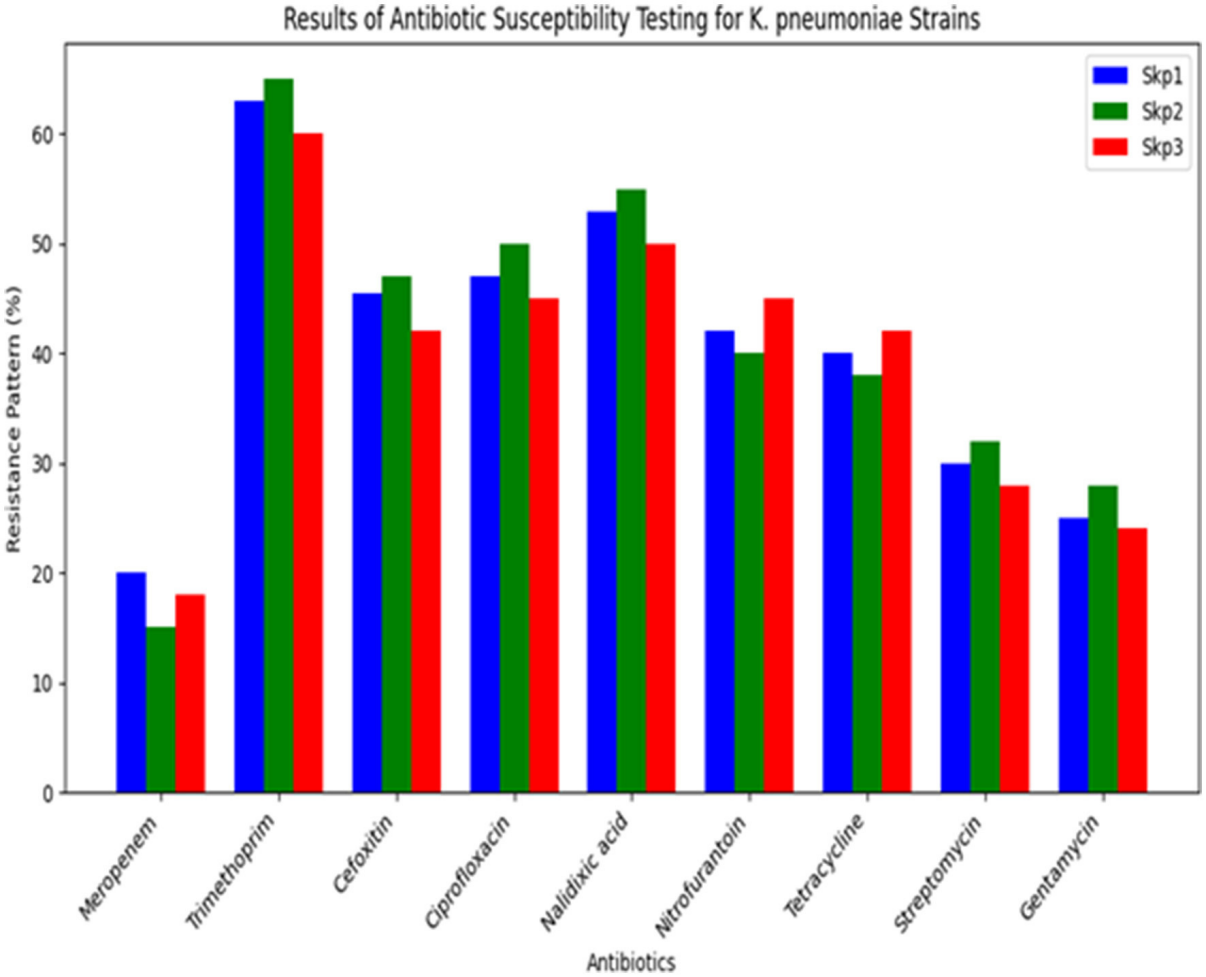
Results of antibiotic susceptibility testing for *K. pneumoniae* strains.

**Figure 15:**
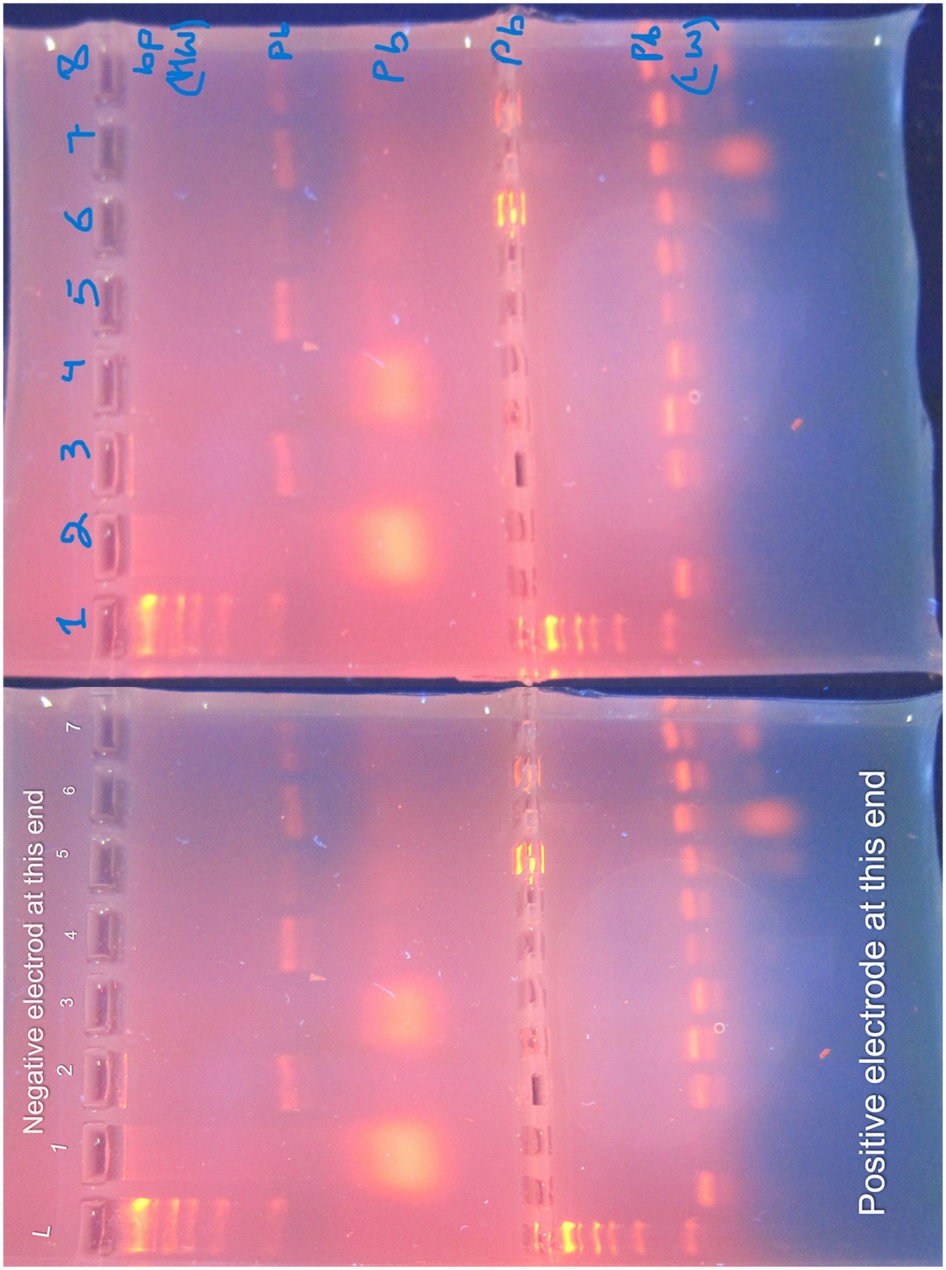
Agarose gel electrophoresis of PCR products. The gel, prepared with 1.5% agarose and stained with ethidium bromide, is visualized under UV light. The DNA ladder (L) is annotated with size markers (in base pairs) to the left of the gel. Lane 1: DNA Ladder with size markers; Lane 2: Positive Control (known *Klebsiella pneumoniae* DNA); Lane 3: Negative Control (PCR reagents without DNA); Lanes 4–6: Samples (identified by separate labels). Heavyweight (HW) bands, representing larger DNA fragments, migrate slower, while lightweight (LW) bands, representing smaller DNA fragments, migrate faster. The gel provides insight into the presence and size of target genes.

The antibiotic susceptibility testing results for different *K. pneumoniae* strains are shown in Table 14. Additionally, Figure 14 provides a visual comparison of these susceptibility patterns across various antibiotics. Finally, the agarose gel electrophoresis results for PCR products are shown in Figure 15. This figure illustrates the presence and size of target genes based on the banding patterns observed in the gel.

**Table 14:** Results of antibiotic susceptibility testing for *K. pneumoniae* strains.

**Antibiotic**	**Resistance pattern (%)**
Meropenem	Skp1 (20), Skp2 (15), Skp3 (18)
Trimethoprim	Skp1 (63), Skp2 (65), Skp3 (60)
Cefoxitin	Skp1 (45.45), Skp2 (47), Skp3 (42)
Ciprofloxacin	Skp1 (47), Skp2 (50), Skp3 (45)
Nalidixic Acid	Skp1 (53), Skp2 (55), Skp3 (50)
Nitrofurantoin	Skp1 (42), Skp2 (40), Skp3 (45)
Tetracycline	Skp1 (40), Skp2 (38), Skp3 (42)
Streptomycin	Skp1 (30), Skp2 (32), Skp3 (28)
Gentamycin	Skp1 (25), Skp2 (28), Skp3 (24)

### 3.5 Gel electrophoresis and PCR amplification

#### 3.5.1 Gel electrophoresis

Agarose gel electrophoresis was performed to visualize the PCR products. The gel was prepared with 1.5% agarose and stained with ethidium bromide. The DNA ladder (molecular weight marker) was included to estimate the size of PCR products. The gel was run at 100 V for 45 min and visualized under UV light. The gel displays heavy weight (HW) bands, which are larger DNA fragments that migrate slower, and light weight (LW) bands, which are smaller fragments that migrate faster.

#### 3.5.2 Controls

To ensure the accuracy of the PCR results, both positive and negative controls were used. The positive control contained DNA from a known *Klebsiella pneumoniae* strain to confirm the presence of the target genes. The negative control, which contained all PCR reagents except DNA, was used to verify the absence of contamination. These controls validate the specificity of the PCR amplification and the accuracy of the gel electrophoresis results.

### 3.6 Summary of ANOVA results

**Figure 16:**
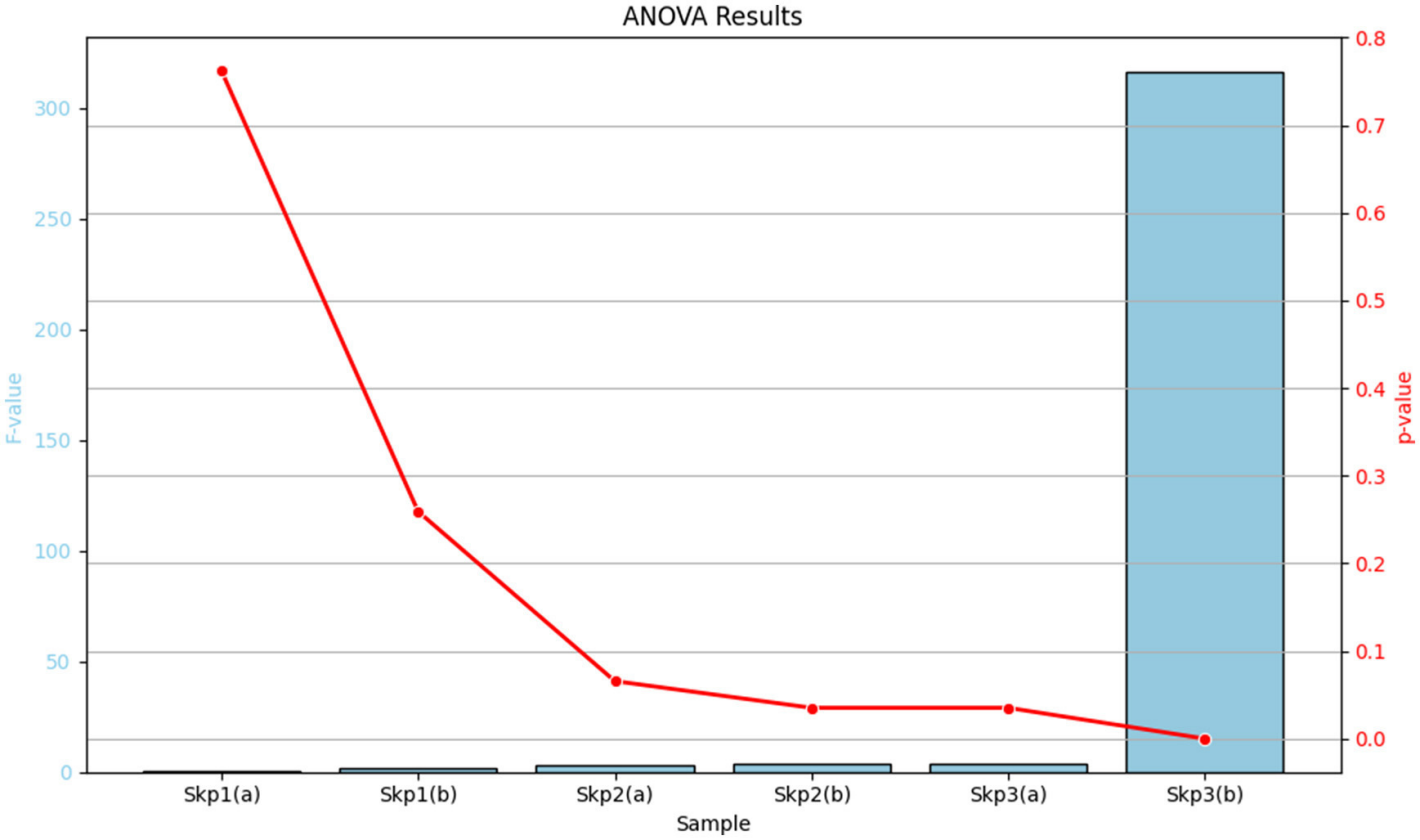
Graphical representation of the ANOVA results. The bar plot illustrates the F-values for each sample, while the line plot depicts the corresponding p-values, highlighting the statistical significance of the results.

The analysis of variance (ANOVA) was performed to evaluate the effects of antibiotics on the zone of inhibition for various samples. The detailed results are summarized in Table 15, which includes the *F*-values and p-values for each sample, along with their significance levels. For a visual representation of these results, Figure 16 displays a bar plot of the *F*-values and a line plot of the *p*-values, providing a clear view of the statistical significance.

**Table 15:** Summary of ANOVA results, detailing the *F*-values and *p*-values for each sample.

**Sample**	***F*-value**	***p*-value**
Skp1(a)	0.5125	0.7620 (non-significant)
Skp1(b)	1.7052	0.2592 (non-significant)
Skp2(a)	3.2832	0.0657 (marginally significant)
Skp2(b)	3.4937	0.0352 (significant)
Skp3(a)	3.4937	0.0352 (significant)
Skp3(b)	315.9481	0.0000 (highly significant)

The *p*-values are annotated with their significance levels.

Table 15 provides a comprehensive tabular summary of the ANOVA results, while Figure 16 offers a visual depiction of these findings, helping to better interpret the statistical significance of the results.

### 3.7 Antibiotic resistance profiles

The antibiotic resistance profiles of *Klebsiella pneumoniae* isolates are comprehensively summarized in Table 16. This table includes the number of isolates that are resistant, intermediate, and sensitive to each antibiotic, as well as the percentage of resistance observed for each antibiotic.

**Table 16:** Summary of antibiotic resistance profiles for *Klebsiella pneumoniae* isolates.

**Antibiotic**	**Number of isolates resistant**	**Number of isolates intermediate**	**Number of isolates sensitive**	**Percentage resistance**
Amoxicillin	15	5	10	50%
Ciprofloxacin	8	3	19	30%
Gentamicin	12	4	14	40%
Tetracycline	10	6	14	40%

The table includes the number of isolates categorized as resistant, intermediate, or sensitive to each antibiotic, along with the percentage of resistance.

**Figure 17:**
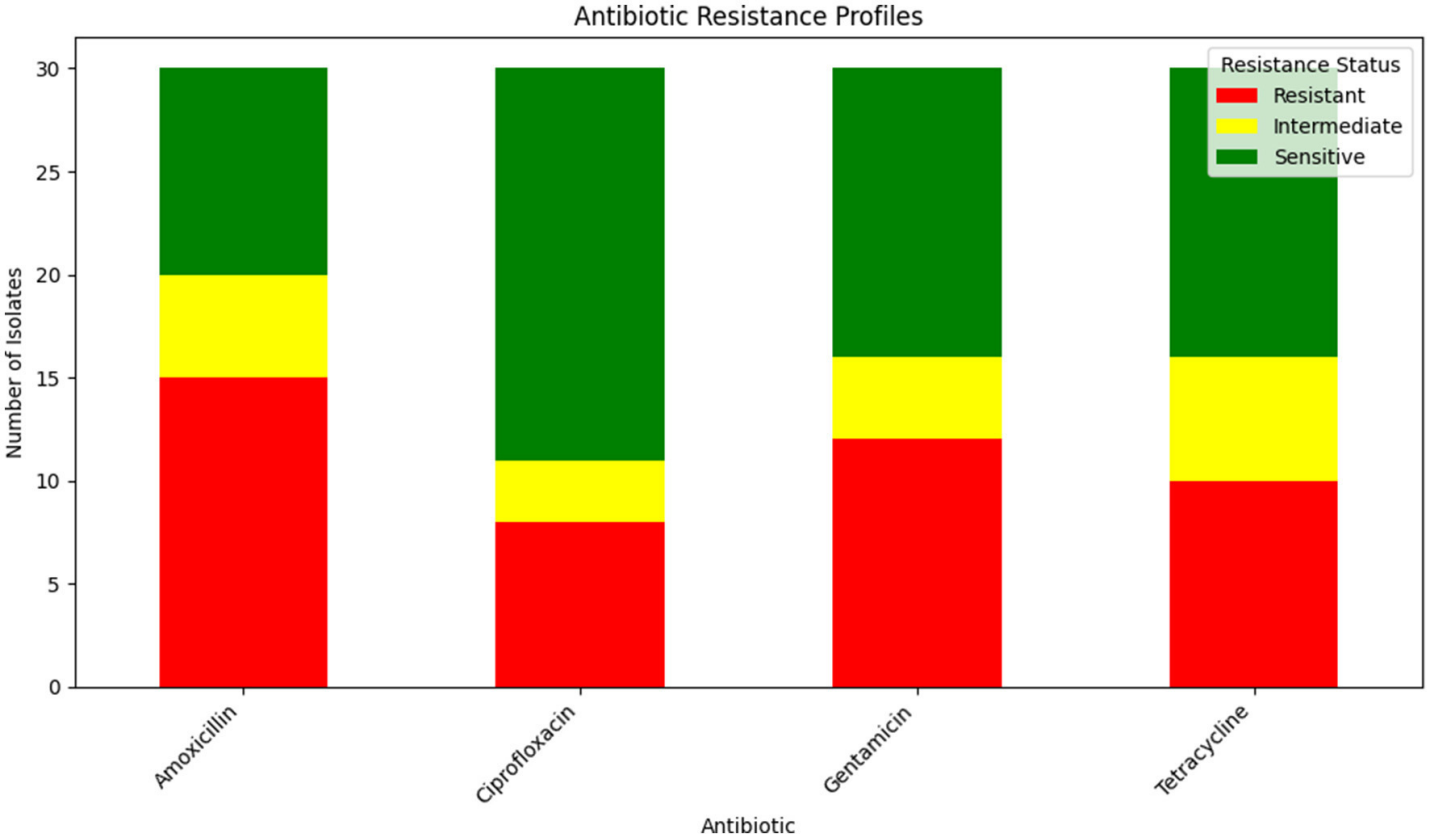
Bar plot showing the distribution of isolates that are resistant, intermediate, and sensitive to each tested antibiotic. This visualization aids in understanding the overall resistance patterns observed in the *Klebsiella pneumoniae* isolates.

For a visual representation of these resistance profiles, Figure 17 provides a bar plot that illustrates the distribution of resistance, intermediate, and sensitive isolates for each tested antibiotic. This visual summary complements the tabular data by presenting a clear overview of the resistance patterns.

Table 16 provides a detailed breakdown of antibiotic resistance profiles, while Figure 17 visually represents these data, highlighting the distribution of resistance, intermediate, and sensitive isolates across the different antibiotics tested.

#### 3.7.1 *Post-hoc* analysis

*Post-hoc* analysis was performed using Tukey's Honest Significant Difference (HSD) test to determine pairwise differences between antibiotic groups. The results of this analysis are detailed in Table 17, which shows the mean differences and p-values for each comparison between antibiotics.

**Table 17:** *Post-hoc* analysis results using Tukey's HSD test.

**Comparison**	**Mean difference**	***p*-value**
Amoxicillin vs. Ciprofloxacin	4.32	0.0001
Amoxicillin vs. Gentamicin	2.18	0.023
Ciprofloxacin vs. Tetracycline	1.67	0.089

The table includes mean differences and *p*-values for pairwise comparisons of antibiotic effectiveness.

**Figure 18:**
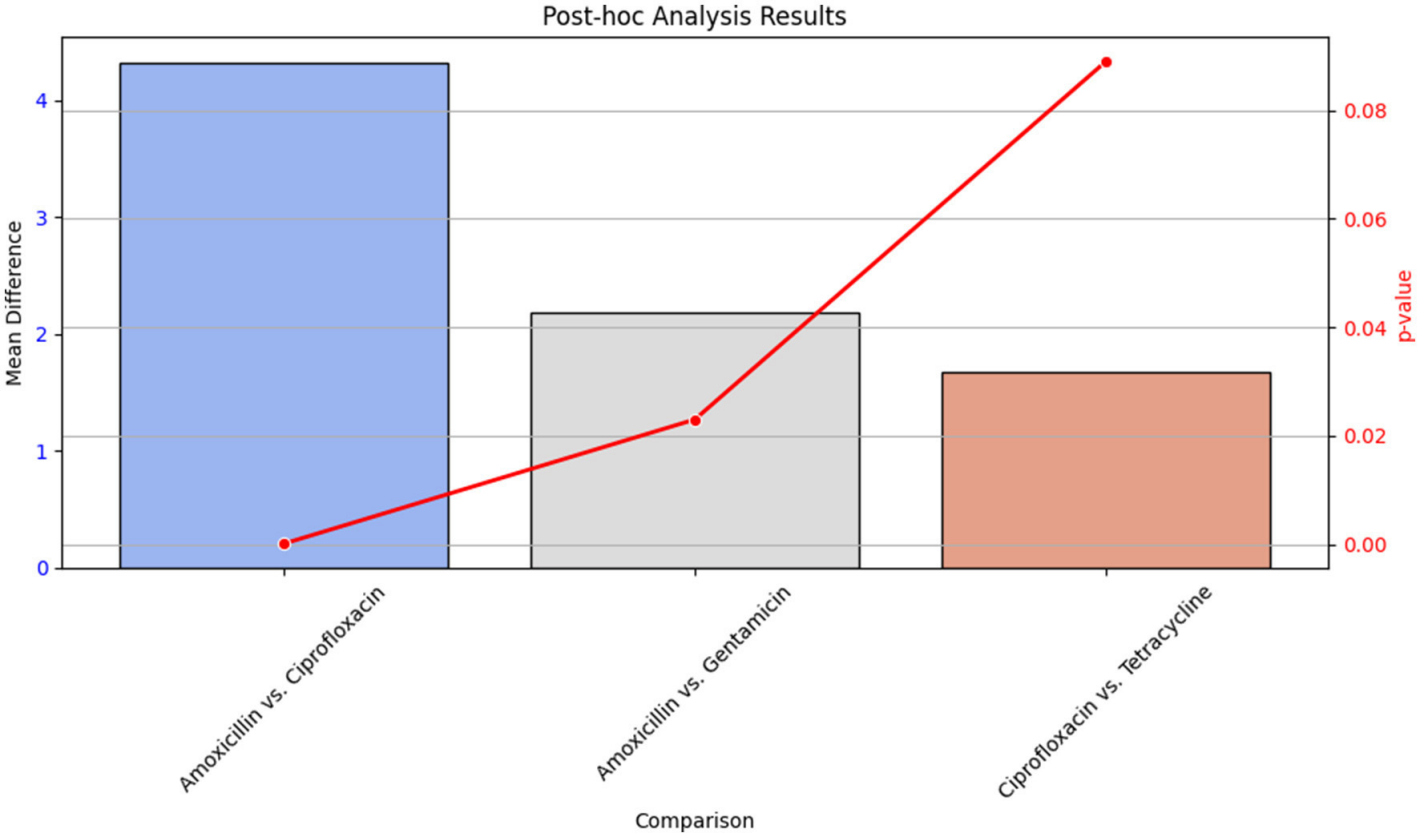
Bar plot showing the mean differences and line plot illustrating p-values from Tukey's HSD test for pairwise comparisons of antibiotic effectiveness. The bar plot highlights significant differences between antibiotics, while the line plot provides the p-values associated with these differences.

Figure 18 provides a visual summary of the *post-hoc* analysis results. The bar plot illustrates the mean differences between antibiotic pairs, while the line plot indicates the corresponding p-values. This visualization helps to highlight which pairwise comparisons showed significant differences in antibiotic effectiveness.

Table 17 presents the statistical outcomes of the pairwise comparisons, and Figure 18 visually depicts these results, offering insights into which antibiotic comparisons revealed significant differences in effectiveness.

#### 3.7.2 Contamination rates

The contamination rates of lettuce samples across different storage durations are summarized in Table 18. This table provides detailed information on the number of samples tested, the number of contaminated samples, and the corresponding contamination rates.

**Table 18:** Contamination rates of lettuce samples.

**Sample age**	**Number of samples tested**	**Number contaminated**	**Contamination rate (%)**
Fresh	30	8	26.7%
2 Days old	30	12	40.0%
4 Days old	30	18	60.0%
1 Week old	30	24	80.0%

The table shows the number of samples tested, the number of contaminated samples, and the contamination rate (%) for each sample age category.

**Figure 19:**
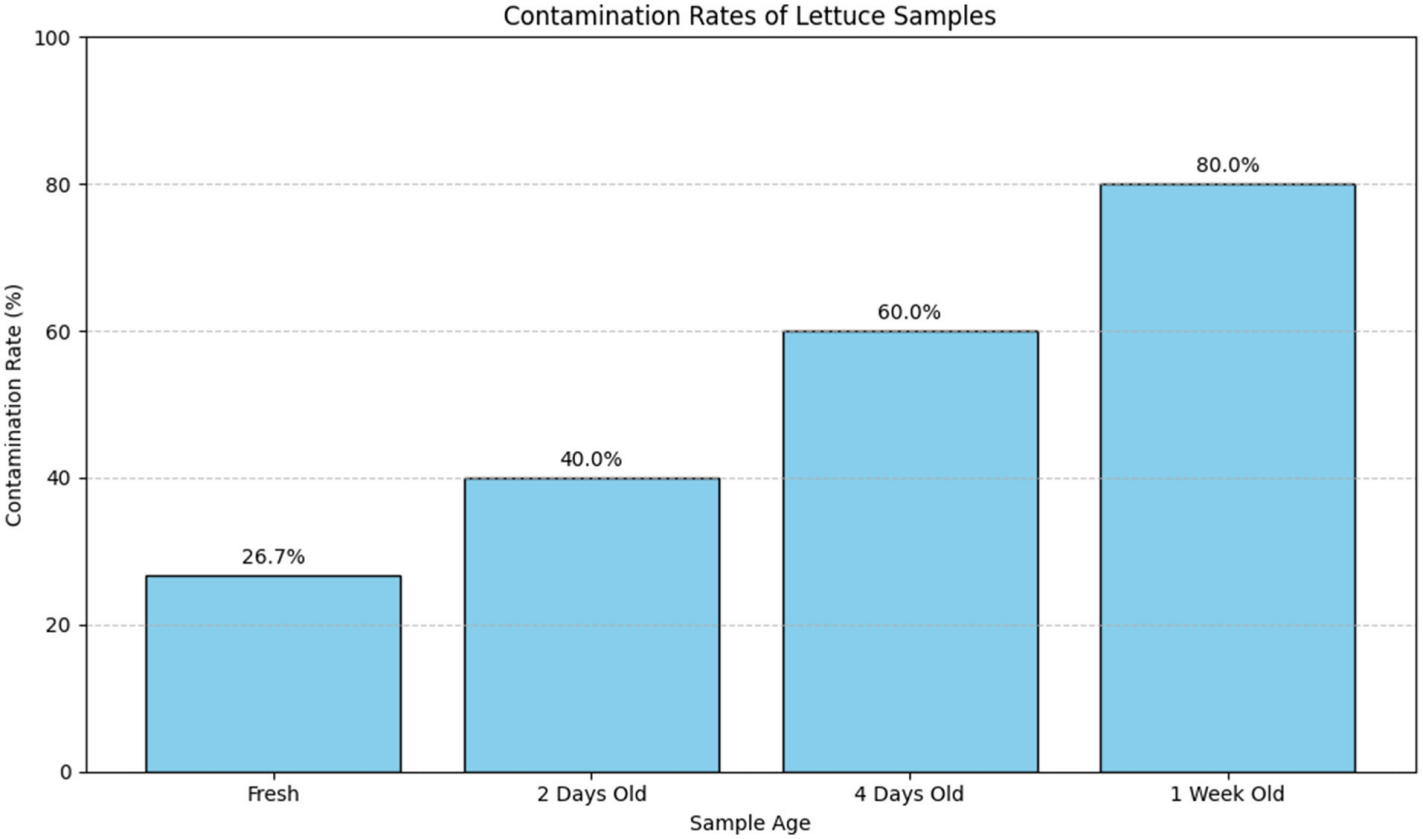
Bar plot illustrating the contamination rates of lettuce samples across different storage durations. The plot shows a clear increase in contamination rates with extended storage time, emphasizing the effect of aging on the contamination levels.

Figure 19 visually represents the contamination rates over the various storage durations. The bar plot provides a clear comparison of contamination rates across different sample ages, highlighting how the rate of contamination increases with longer storage times.

Table 18 provides a detailed numerical summary of the contamination rates, while Figure 19 visually demonstrates the trend of increasing contamination with longer storage times, offering a more intuitive understanding of the data.

### 3.8 Mathematical model for ANOVA results

#### 3.8.1 Model assumptions

In our study, linear regression was utilized to further analyze the results from ANOVA. The linear regression model assumes that the response variable, Zone of Inhibition (*Y*), follows a normal distribution within each category of the predictor variable, antibiotics (*X*). Additionally, the model presumes homoscedasticity, meaning that the variance of *Y* is constant across all levels of *X*. These assumptions are essential for ensuring the validity of the regression analysis and for accurately interpreting the results obtained from ANOVA.

#### 3.8.2 Description of variables and parameters

The following variables and parameters are defined within the linear regression model:

- *Y*: Zone of Inhibition, which measures the diameter of the bacterial growth inhibition zone around the antibiotic disc.
- *X*: Antibiotics, the independent variable representing different types of antibiotics used.
- β_0_: Intercept, representing the estimated baseline Zone of Inhibition in the absence of antibiotics.
- β_1_: Coefficient for *X*, indicating the change in Zone of Inhibition associated with each unit change in the concentration of antibiotics.
- ϵ: Error term, which captures the unexplained variability in the response variable.

#### 3.8.3 Model equations

The linear regression model used to extend the ANOVA results is expressed as:


(1) $$\begin{array}{l} Y=\beta_{0}+\beta_{1}X+\epsilon \end{array}$$

In this equation, *Y* is the dependent variable (Zone of Inhibition), *X* is the independent variable (type of antibiotic), β_0_ is the intercept, β_1_ is the coefficient for *X*, and ϵ represents the error term. This regression model helps quantify how the Zone of Inhibition varies with changes in antibiotic concentration, providing a more detailed analysis beyond the scope of ANOVA alone.

#### 3.8.4 Fitting simulated data

Simulated data were generated to test the robustness of the linear regression model. To simulate the data, we used the parameter estimates from our initial ANOVA analysis and created synthetic datasets with varying antibiotic concentrations. The simulation involved generating random data points based on the assumed normal distribution and homoscedasticity, incorporating both the estimated parameters and random error. This simulated data was then used to fit the linear regression model, providing a means to evaluate the model's predictive performance and robustness.

#### 3.8.5 Parameter values and discussion

The estimated parameters from the linear regression model are:

- β_0_ = 10.67: Represents the estimated Zone of Inhibition in the absence of antibiotics, serving as a baseline measurement.
- β_1_ = 0.46: Indicates the average change in the Zone of Inhibition per unit change in antibiotic concentration, suggesting a positive correlation between antibiotic presence and inhibition zone.

These parameters were derived using ordinary least squares regression, which minimizes the sum of squared residuals to fit the model to the data.

#### 3.8.6 Basic information about model implementation

The linear regression model was implemented using statistical software in Python, utilizing libraries such as NumPy and SciPy. Ordinary least squares regression was employed to estimate the model parameters. ANOVA was used initially to identify significant differences among antibiotic treatments, while linear regression provided a detailed quantitative assessment of how antibiotic concentration impacts the Zone of Inhibition.

#### 3.8.7 Sample code

A Python code snippet demonstrating the calculation of model coefficients and the integration with ANOVA results is included in the Supplementary material. This code illustrates how the statistical methods were applied to derive and interpret the model parameters.

### 3.9 Explanation of the figure and sample simulation results

**Figure 20:**
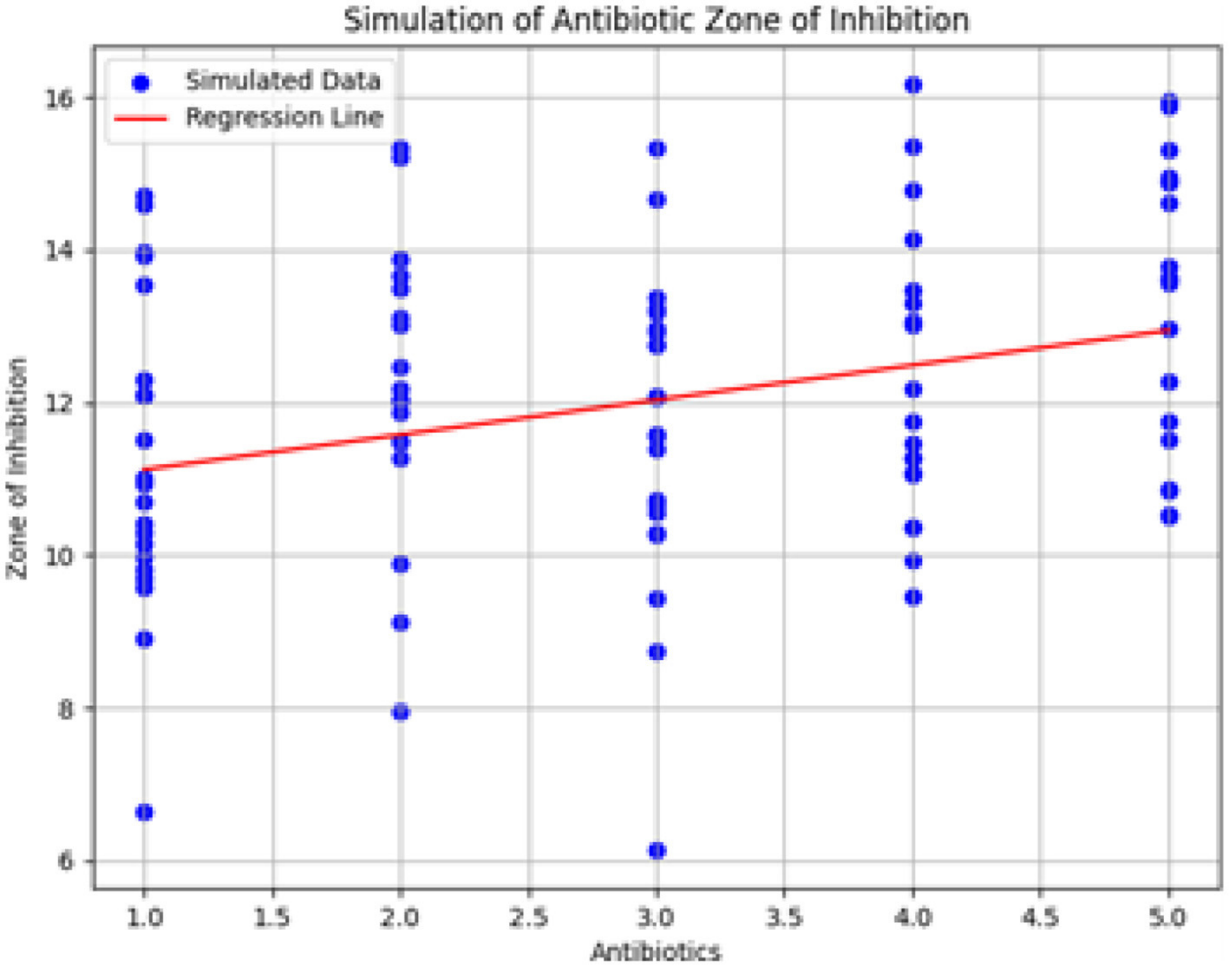
Simulation of antibiotic zone of inhibition based on linear regression. The figure illustrates the predicted relationship between antibiotic types and the Zone of Inhibition, highlighting the regression line and the associated data variability.

Figure 20 shows the simulation of the Zone of Inhibition based on the linear regression model, following ANOVA analysis. The x-axis represents different types of antibiotics, and the y-axis shows the predicted Zone of Inhibition. The red line denotes the regression line derived from the model, while the scatter of blue dots represents the observed data points, reflecting the variability captured by the error term.

#### 3.9.1 Additional simulation results

The simulation results include:

- Predicted Zones of Inhibition for various antibiotics, demonstrating the model's fit.
- Visualization of the regression line relative to the data points.
- Confidence intervals for the model coefficients, offering insights into the precision and reliability of the estimated parameters.

### 3.10 Discussion of simulation results

The simulation results provide insights into the relationship between antibiotics and the Zone of Inhibition. By examining the model coefficients and their confidence intervals, we gain a clearer understanding of how different antibiotics affect bacterial growth inhibition. This analysis enhances the interpretation of antibiotic efficacy and contributes to a more detailed assessment of treatment options.

## 4 Discussion

Salads, including lettuce leaves, are considered to be a rich source of various nutrients, including vitamin A, vitamin C, calcium, beta carotene, and folate (Oliveira et al., 2020). However, in the current study, it was found that lettuce leaves, which are commonly consumed daily, have a high load of gram-negative bacteria. A higher bacterial load poses a greater threat to consumers (Glynn et al., 2006). The presence of *Klebsiella* as a microbiota on lettuce has also been reported in South Korea (Alemu et al., 2018).

The findings of this study highlight significant public health and food safety concerns regarding the prevalence of *Klebsiella pneumoniae* on lettuce leaves, a commonly consumed vegetable. Lettuce, valued for its nutritional benefits, including essential vitamins and minerals, is often featured in salads and other dishes. However, our observation of a 50% contamination rate with *Klebsiella pneumoniae* underscores a serious issue. The presence of this gram-negative bacterium on such a frequently consumed food item raises critical concerns about foodborne illness risks and the potential for widespread contamination.

### 4.1 Implications for public health and food safety

*Klebsiella pneumoniae* is an opportunistic pathogen that can cause a range of infections, from respiratory and urinary tract infections to more severe conditions. The high contamination rate of 50% found in this study indicates a substantial risk of exposure to this pathogen for consumers of lettuce in the study area. This is particularly troubling given the bacterium's resistance to multiple antibiotics, which complicates treatment options for infections resulting from contaminated food sources.

Antibiotic resistance in *Klebsiella pneumoniae* is an escalating global health issue. Our study found resistance to several antibiotics, including doxycycline, ticarcillin, and vancomycin, highlighting the challenges in managing infections caused by this bacterium. The high resistance rates, combined with susceptibility to gentamicin, underscore the need for effective antimicrobial stewardship and continuous monitoring of resistance patterns to safeguard public health.

### 4.2 Comparison with similar studies

Our study reveals a 50% contamination rate of *Klebsiella pneumoniae* in lettuce, which provides valuable insights when compared to other research findings. This section elaborates on the comparison with similar studies to explore possible reasons for the observed variations.

Calbo et al. (2011) conducted a study in Spain, finding a 35% colonization rate of *Klebsiella pneumoniae* on hospital kitchen surfaces and food items (Calbo et al., 2011). Although their study focuses on hospital settings, which generally maintain stringent hygiene standards, their reported prevalence is lower than our 50% contamination rate. This discrepancy may be attributed to the rigorous cleaning protocols and infection control measures in hospitals, which are likely more effective than those typically employed in general consumer environments. Furthermore, the study's focus on hospital settings may not directly translate to the general public's food handling practices.

Conversely, Romyasamit et al. (2021) identified an ESBL-producing *Klebsiella pneumoniae* prevalence of only 4.6% in food samples from Thailand (Romyasamit et al., 2021). This significantly lower prevalence compared to our findings could reflect more effective food safety practices in Thailand, including stringent sanitation protocols and antibiotic stewardship programs. The lower contamination rates in Thailand might also be related to differences in agricultural practices, environmental conditions, and public health measures aimed at controlling bacterial contamination in food products.

A study from Andhra Pradesh, India, reported a notably higher contamination rate of 71.12% in various food samples, with *Klebsiella pneumoniae* accounting for 40% of these cases (Santajit and Indrawattana, 2016). This higher prevalence underscores significant regional variations in contamination levels and may indicate less effective food safety measures or higher levels of environmental contamination. The variation in contamination rates highlights the need for localized interventions and improved food safety practices tailored to regional conditions.

Theocharidi et al. (2022) found a high prevalence of *Klebsiella pneumoniae* in Greek meat products, with significant detection of virulence and antimicrobial resistance genes (Theocharidi et al., 2022). Their study, though focusing on meat products rather than vegetables, reported a contamination rate of up to 25%, which is lower than our 50% prevalence but still indicates a significant concern. The presence of antimicrobial resistance genes in their study further emphasizes the public health implications of *Klebsiella pneumoniae* contamination. Differences in contamination rates between our study and Theocharidi et al.'s research may be influenced by the types of food products studied, regional practices, and the specific methodologies employed. These findings align more closely with our results compared to the lower prevalence observed in Thailand. The similarities between these studies suggest that regions with comparable food handling practices and environmental conditions may experience similar contamination levels. However, the variability across different studies indicates that local factors such as hygiene practices and regulatory measures significantly impact contamination rates.

Crippa et al. (2023) reported genomic features of *Klebsiella* isolates from artisanal ready-to-eat food production facilities, finding notable prevalence rates in such settings (Crippa et al., 2023). Although this study primarily focuses on genomic aspects and artisanal food production, it provides insights into the broader context of *Klebsiella pneumoniae* prevalence in food environments. The findings suggest that contamination rates in artisanal food production can be significant, potentially reflecting practices that may vary from industrial or large-scale production settings. This comparison with our study highlights that contamination levels can vary depending on food production practices and settings.

These comparisons illustrate the complex nature of foodborne pathogen contamination and the influence of diverse factors such as regional hygiene practices, environmental conditions, and methodological differences. The higher contamination rate observed in our study compared to some reports indicates potential weaknesses in current food safety practices or environmental controls within the study area. In contrast, the higher contamination rates reported in studies from regions like India demonstrate that contamination can be highly variable and context-dependent. This variability underscores the importance of region-specific research and interventions to address and mitigate contamination risks effectively.

Future research should focus on standardized methodologies, larger sample sizes, and cross-regional studies to provide a clearer understanding of contamination variations. Implementing rigorous monitoring, enhancing food safety practices, and developing tailored interventions based on regional conditions are essential steps in reducing the risk of contamination and protecting public health.

The higher contamination rate observed in our study compared to some reports indicates potential weaknesses in current food safety practices or environmental controls within the study area. In contrast, the higher contamination rates reported in studies from regions like India demonstrate that contamination can be highly variable and context-dependent. This variability underscores the importance of region-specific research and interventions to address and mitigate contamination risks effectively.

### 4.3 Study limitations

Several limitations must be considered in interpreting these results. The sample size of 120 lettuce samples, though substantial, may not fully represent variability across different geographic locations or seasonal changes. The focus on Risalpur may limit the applicability of the findings to other regions with different environmental and agricultural practices. Additionally, potential biases in sample collection and analysis, such as variations in handling procedures and laboratory techniques, could influence the accuracy and generalizability of the results.

### 4.4 Suggestions for future research

Future research should address these limitations by including larger and more diverse sample sizes across various regions and seasons to provide a more comprehensive understanding of contamination patterns and antibiotic resistance. Future studies should also explore intervention strategies to reduce contamination and antibiotic resistance, including:

- **Improving hygiene practices:** Implementing and enforcing better hygiene and sanitation practices during food handling, storage, and transportation to minimize contamination.
- **Safe water use:** Ensuring the use of clean, uncontaminated water for irrigation and washing vegetables to reduce the risk of bacterial contamination.
- **Education and training:** Providing education and training for food handlers, vendors, and consumers on proper food safety measures and the importance of hygiene.
- **Disinfection methods:** Evaluating the effectiveness of various disinfection and sanitization methods to reduce bacterial loads and prevent the spread of antibiotic-resistant pathogens.
- **Monitoring resistance trends:** Continuously monitoring antibiotic resistance trends in foodborne pathogens to inform public health policies and practices.

In summary, this study reveals a significantly higher rate of *Klebsiella pneumoniae* contamination in lettuce leaves in Risalpur. The presence of these pathogenic gram-negative bacteria poses a substantial risk for transmitting enteric pathogens through the food chain. Contributing factors may include poor management, improper transportation and storage of food in unhygienic conditions, and the use of contaminated water for irrigation and vegetable spraying.

The obtained ANOVA results provide insights into the relationship between different antibiotics and their impact on the Zone of Inhibition, an indicator of antibiotic susceptibility. The mathematical model developed using linear regression expresses the Zone of Inhibition (*Y*) as a function of antibiotic concentration (*X*) and includes an error term (ε). The model equation, *Y* = β_0_+β_1_*X*+ε, with coefficients β_0_ = 10.67 and β_1_ = 0.46, indicates that the baseline Zone of Inhibition without antibiotics is 10.67 mm, and it increases by 0.46 mm for each unit increase in antibiotic concentration. The ANOVA results showed an *F*-value of 0.4783 and a *p*-value of 0.7620, suggesting that the observed relationship was not statistically significant at the 0.05 level. This may reflect sample size limitations or data variability.

Overall, the mathematical model and ANOVA results contribute to understanding antibiotic susceptibility in *Klebsiella pneumoniae* strains isolated from raw vegetables. Continued research is essential to investigate factors influencing antibiotic resistance and develop targeted interventions to control the spread of resistant pathogens in the food chain.

## 5 Summary

- The study aimed to assess the antibiotic susceptibility of bacterial isolates from various samples.
- An antibiotic susceptibility test was conducted using 16 types of antibiotic disks. This revealed diverse levels of susceptibility, resistance, and intermediate activity against different antibiotics.
- The researchers employed a linear regression-based mathematical model to summarize the ANOVA results for the antibiotic susceptibility test. This provided valuable insights into antimicrobial activity.
- The findings highlighted the presence of antibiotic-resistant strains. Notably, resistance to doxycycline, ticarcillin, and vancomycin was observed in the tested samples.
- The isolates showed high susceptibility to gentamicin, indicating the presence of multidrug-resistant strains. This underscores the need for effective antibiotic stewardship strategies.
- Research on ESBL-producing *Klebsiella pneumoniae* isolates from raw vegetables and ready-to-eat food revealed similar variations in antibiotic resistance. This emphasizes the need for strict antibiotic stewardship programs and hygiene practices.
- In conclusion, the study highlights the importance of prudent antibiotic use and stringent hygiene measures to combat the growing threat of antibiotic resistance. Effective measures are crucial to safeguard public health.
- Continuous research and surveillance efforts are vital to address emerging antibiotic resistance trends and their impact on global health.

## 6 Conclusion

This study reveals a notable prevalence of *Klebsiella pneumoniae* and other gram-negative bacteria in raw vegetables, with lettuce being particularly affected. These findings underscore a critical public health issue, emphasizing the necessity for enhanced hygiene practices throughout the food handling and storage processes.

Based on our findings, we propose the following recommendations:

1. **Strengthen food hygiene practices:** It is imperative to implement rigorous hygiene protocols at all stages of food production, from cultivation to consumption. This includes regular sanitation training for food handlers and stringent cleaning procedures in food processing facilities.
2. **Enhance surveillance and testing:** Establish comprehensive surveillance systems to monitor bacterial contamination and antibiotic resistance in food products. Regular and systematic testing should be employed to detect and address contamination issues promptly.
3. **Promote antibiotic stewardship:** Develop and enforce policies aimed at optimizing the use of antibiotics in both human healthcare and agriculture. Effective antibiotic stewardship is essential to mitigate the spread of resistant strains and preserve the efficacy of existing antibiotics.
4. **Increase public and professional awareness:** Educate consumers, food producers, and healthcare professionals about the risks associated with antibiotic-resistant pathogens and the importance of maintaining high standards of food safety. Awareness campaigns should focus on best practices for food handling and resistance prevention.
5. **Support ongoing research:** Invest in research initiatives that explore the mechanisms of antibiotic resistance and develop novel intervention strategies. Collaborative research efforts are crucial to advancing our understanding and control of antibiotic resistance in foodborne pathogens.

Addressing these recommendations will be pivotal in mitigating the risks associated with antibiotic-resistant pathogens and enhancing overall public health safety. Adopting a One Health approach, which integrates human, animal, and environmental health, is essential for effective management and reduction of antibiotic resistance. Through concerted efforts in these areas, we can work toward a sustainable future with minimized impacts from antibiotic-resistant pathogens.

## 7 Limitations

This study has several limitations:

- The focus on lettuce in a single region limits the generalizability of the findings to other regions and types of vegetables.
- The sample size is not specified, which affects the reliability and statistical power of the results.
- There is no control group or comparison to other vegetables or non-contaminated samples, which limits the ability to draw broader conclusions about contamination levels and sources.

## Data Availability

The original contributions presented in the study are included in the article/Supplementary material, further inquiries can be directed to the corresponding author.
