# Supplementary material for: Isolation and characterization of pathogenic Klebsiella pneumoniae strains from lettuce: a potential source of antibiotic resistance and development of a mathematical model for ANOVA results
Source: Front Microbiol. 2024 Sep 24;15:1473055. doi: 10.3389/fmicb.2024.1473055 (PMC11459608; doi:10.3389/fmicb.2024.1473055)
Supplement: Supplementary file 1 [file Data_Sheet_1.PDF]

supplementary tables

ruby.khan

March 2024

1 Supplementary tables and Figures

2 Suppl Figure

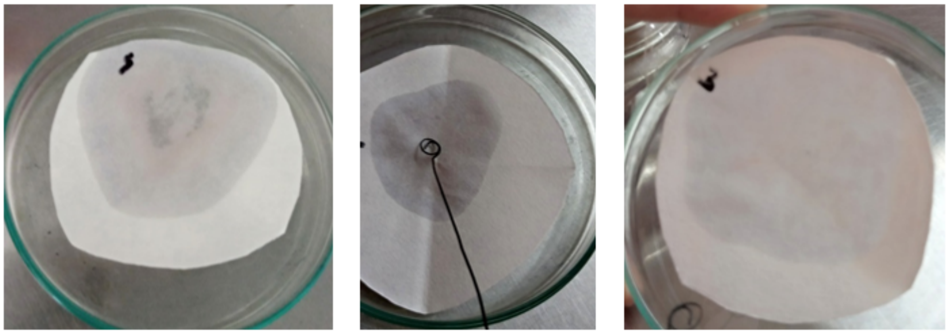

Figure 1: Visualization of Oxidase Test Results

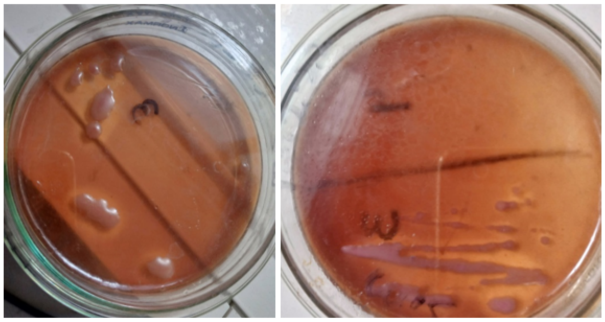

Figure 2: Colony appearance on MacConkey agar

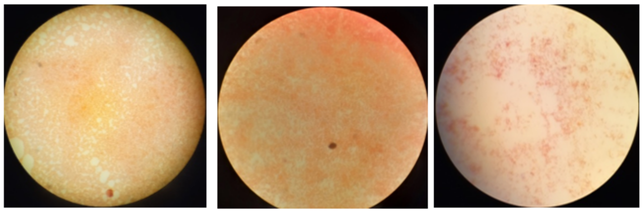

Figure 3: Gram-negative isolates of *Klebsiella pneumoniae*

Python Code

```
import numpy as np
import matplotlib.pyplot as plt
```

Chemicals and Equipment Used During the Research
Gram iodine solution
Crystal violet dye
Safranin
Decolorizer
Nutrient broth
Nutrient agar
Tryptic soya agar
MacConkey agar
Muller Hingston agar
Andrade indicator
Kovac reagent
Hydrogen peroxide
Different sugars
Oil immersion
Antibacterial drugs
Forceps
Digital balance
Biosafety cabinet
Incubator
Microscope
Autoclave
Refrigerator
Mortar and pestle
Glass slides
Filter paper
Conical flask
Test tubes
Durham's tubes
Inoculation loop
Spirit lamp
Pipette
Petri dishes
Graduated cylinder
Beakers
Funnels
Distilled water
Aluminum foil

Table 1: List of Chemicals and Equipment Utilized in the Research

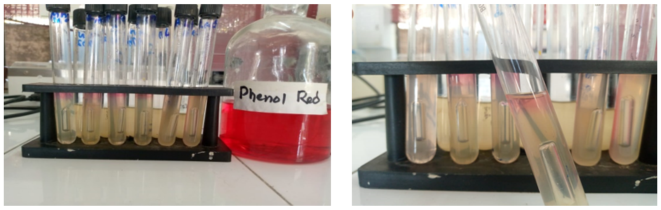

Figure 4: Sugar Fermentation Positive Bacterial Isolates

S.No	Antibiotics	Abbreviation	Concentration (mg/ml)	Company	Disc Potency (µg)
1	Vancomycin	VA	10	Merck Pakistan	30
2	Amoxicillin+Clavulante	AMC	20	Merck Pakistan	30
3	Ceftriaxone	CRO	30	Merck Pakistan	30
4	Gentamicin	CN	40	Merck Pakistan	30
5	Meropenem	MEM	5	Merck Pakistan	10
6	Imipenem	IPM	6	Merck Pakistan	10
7	Chloramphenicol	C	7	Merck Pakistan	30
8	Doxycycline	DO	8	Merck Pakistan	30
9	Amikacin	AK	9	Merck Pakistan	30
10	Piperacillin	PRL	10	Merck Pakistan	100
11	Ticarcilline	TIC	11	Merck Pakistan	75
12	Polymixin B	PB	12	Merck Pakistan	300
13	Cefepime	FEP	13	Merck Pakistan	30
14	Tazobactam Piperacillin	TZP	14	Merck Pakistan	-
15	Ciprofloxacin	CIP	15	Merck Pakistan	5
16	Ampicillin	AMP	16	Merck Pakistan	10

Table 2: Antibiotics List with Concentration, Company Name, and Disc Potency for Laboratory Testing

S.NO	Strains	Gram Staining
1	Skp1	Negative
2	Skp2	Negative
3	Skp3	Negative

Table 3: Gram Staining of Bacterial Isolates

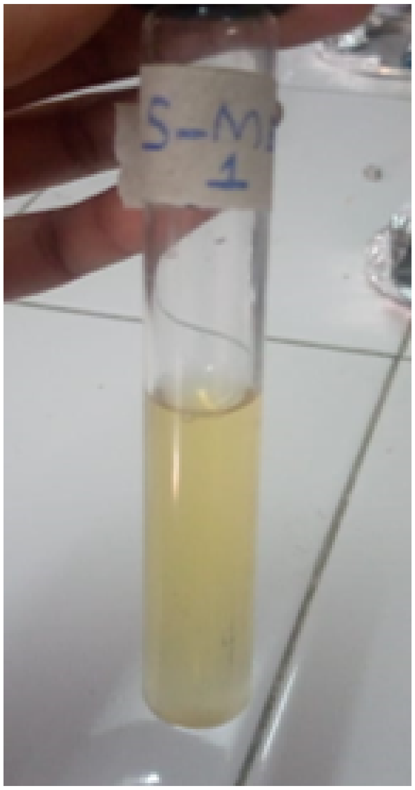

Figure 5: Indole Negative Bacterial Isolates

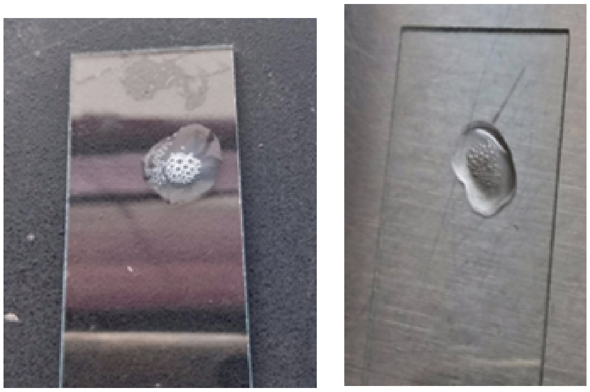

Figure 6: Catalase Positive Bacterial Isolates

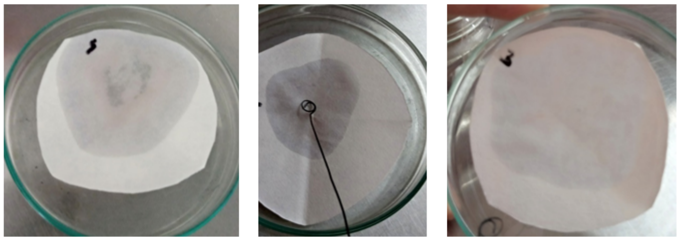

Figure 7: Oxidase Negative Bacterial Isolates

```
# Define the model coefficients
beta_0 = 10.6667
beta_1 = 0.4556

# Generate sample data for Antibiotics (X)
num_samples = 100
antibiotics_levels = np.random.randint(1, 6, size=num_samples)
# Randomly select antibiotic levels

# Simulate Zone of Inhibition (Y) based on the model equation
error = np.random.normal(0, 2, size=num_samples)
# Simulate random error
zone_of_inhibition = beta_0 + beta_1 * antibiotics_levels + error

# Plot the simulated data
plt.figure(figsize=(8, 6))
plt.scatter(antibiotics_levels, zone_of_inhibition, color=’blue’, label=’Simulated Data’)

plt.xlabel(’Antibiotics’)
plt.ylabel(’Zone of Inhibition’)
plt.title(’Simulation of Antibiotic Zone of Inhibition’)
plt.grid(True)

# Plot the regression line
x_values = np.linspace(1, 5, 100)
y_values = beta_0 + beta_1 * x_values
plt.plot(x_values, y_values, color=’red’, label=’Regression Line’)

plt.legend()
plt.show()
```
